# Supplementary material for: A resilience group training program for people with multiple sclerosis: Results of a pilot single-blind randomized controlled trial and nested qualitative study
Source: PLoS One. 2020 Apr 9;15(4):e0231380. doi: 10.1371/journal.pone.0231380 (PMC7145197; doi:10.1371/journal.pone.0231380)
Supplement: S1 Appendix — (PDF) [file pone.0231380.s001.pdf]

S1 Appendix - CONSORT

Checklist of information to include when reporting a pilot or feasibility trial

Section/Topic	Item No	Checklist item	Reported on page No
**Title and abstract**			
	1a	Identification as a pilot or feasibility randomised trial in the title	Title page, p. 1
	1b	Structured summary of pilot trial design, methods, results, and conclusions	Abstract p. 2,3
**Introduction**			
Background and objectives	2a	Scientific background and explanation of rationale for future definitive trial, and reasons for randomised pilot trial	Introduction p. 4-7
	2b	Specific objectives or research questions for pilot trial	Introduction p. 6-7
**Methods**			
Trial design	3a	Description of pilot trial design (such as parallel, factorial) including allocation ratio	Materials and methods p. 7, and Recruitment and trial procedures p. 8,9
	3b	Important changes to methods after pilot trial commencement (such as eligibility criteria), with reasons	N/A
Participants	4a	Eligibility criteria for participants	Participant eligibility p. 8
	4b	Settings and locations where the data were collected	Material and Methods p. 8
	4c	How participants were identified and consented	Recruitment and trial procedures p. 8, 9.

Interventions	5	The interventions for each group with sufficient details to allow replication, including how and when they were actually administered	Intervention p. 9; Control condition p. 10
Outcomes	6a	Completely defined prespecified assessments or measurements to address each pilot trial objective specified in 2b, including how and when they were assessed	Material and Methods p. 7; Measures p. 10 – 13.
	6b	Any changes to pilot trial assessments or measurements after the pilot trial commenced, with reasons	N/A
	6c	If applicable, prespecified criteria used to judge whether, or how, to proceed with future definitive trial	
Sample size	7a	Rationale for numbers in the pilot trial	Sample size calculation p. 14; S4 Appendix – Sample size calculation.
	7b	When applicable, explanation of any interim analyses and stopping guidelines	N/A
Randomisation:			
Sequence generation	8a	Method used to generate the random allocation sequence	Recruitment and trial procedures p. 8, 9.
	8b	Type of randomisation(s); details of any restriction (such as blocking and block size)	Recruitment and trial procedures p. 9.
Allocation concealment mechanism	9	Mechanism used to implement the random allocation sequence (such as sequentially numbered containers), describing any steps taken to conceal the sequence until interventions were assigned	Recruitment and trial procedures p. 9
Implementation	10	Who generated the random allocation sequence, who enrolled participants, and who assigned participants to interventions	Recruitment and trial procedures p. 8, 9.
Blinding	11a	If done, who was blinded after assignment to interventions (for example, participants, care providers, those assessing	Material and

		outcomes) and how	Methods p. 7
	11b	If relevant, description of the similarity of interventions	Discussion p.34
Statistical methods	12	Methods used to address each pilot trial objective whether qualitative or quantitative	Quantitative Analysis p. 14; Qualitative approach p. 15.
**Results**			
Participant flow (a diagram is strongly recommended)	13a	For each group, the numbers of participants who were approached and/or assessed for eligibility, randomly assigned, received intended treatment, and were assessed for each objective	Fig. 1 - CONSORT flowchart.
	13b	For each group, losses and exclusions after randomisation, together with reasons	Fig. 1 - CONSORT flowchart.; Results/ RCT findings/partici pants characteristics p. 16.
Recruitment	14a	Dates defining the periods of recruitment and follow-up	Results/ RCT findings/partici pants characteristics p. 16.
	14b	Why the pilot trial ended or was stopped	N/A
Baseline data	15	A table showing baseline demographic and clinical characteristics for each group	Table 1 and 2 p 16 – 18.
Numbers analysed	16	For each objective, number of participants (denominator) included in each analysis. If relevant, these numbers should be by randomised group	We had no drop out. N= 37
Outcomes and estimation	17	For each objective, results including expressions of uncertainty (such as 95% confidence interval) for any estimates. If relevant, these results should be by randomised group	Efficacy results p. 17-19
Ancillary analyses	18	Results of any other analyses performed that could be used to inform the future definitive trial	Efficacy results

			p. 17-19.<br>S5 Appendix – Graphic plots for repeated measure analysis
Harms	19	All important harms or unintended effects in each group (for specific guidance see CONSORT for harms)	N/A
	19a	If relevant, other important unintended consequences	N/A
**Discussion**			
Limitations	20	Pilot trial limitations, addressing sources of potential bias and remaining uncertainty about feasibility	Discussion p. 33 - 37.
Generalisability	21	Generalisability (applicability) of pilot trial methods and findings to future definitive trial and other studies	Discussion p. 33 - 37.
Interpretation	22	Interpretation consistent with pilot trial objectives and findings, balancing potential benefits and harms, and considering other relevant evidence	Discussion p. 33 - 37.
	22a	Implications for progression from pilot to future definitive trial, including any proposed amendments	Discussion p. 33 - 37.
**Other information**			
Registration	23	Registration number for pilot trial and name of trial registry	Materials and methods p. 7: ISRCTN registration number: 38971970
Protocol	24	Where the pilot trial protocol can be accessed, if available	Materials and methods p. 7; ISRCTN website
Funding	25	Sources of funding and other support (such as supply of drugs), role of funders	Funding p. 38
	26	Ethical approval or approval by research review committee, confirmed with reference number	Matherials and methods p. 8
