# Supplementary material for: A resilience group training program for people with multiple sclerosis: Results of a pilot single-blind randomized controlled trial and nested qualitative study
Source: PLoS One. 2020 Apr 9;15(4):e0231380. doi: 10.1371/journal.pone.0231380 (PMC7145197; doi:10.1371/journal.pone.0231380)
Supplement: S2 Appendix — (PDF) [file pone.0231380.s002.pdf]

S2 Appendix - COREQ

Consolidated criteria for reporting qualitative studies (COREQ): 32-item checklist

No. Item	Guide questions/description	Reported on Page #
**Domain 1: Research team and reflexivity**		
Personal Characteristics		
1. Inter viewer/facilitator	Which author/s conducted the inter view or focus group?	
2. Credentials	What were the researcher's credentials? E.g. PhD, MD	Rui Quintas: MSc
3. Occupation	What was their occupation at the time of the study?	Qualitative study participants and recruitment p. 14
4. Gender	Was the researcher male or female?	Male
5. Experience and training	What experience or training did the researcher have?	Qualitative study participants and recruitment p. 14<br><br>He previously participated in a dedicated training for another project involving people with MS (ManTra project). He has already run PSIs and FGMs and performed qualitative data analysis.
Relationship with participants		
6. Relationship established	Was a relationship established prior to study commencement?	The interviewer was not acquainted to the participants prior to study commencement. When the interviews were conducted, the interviewer had already met the participants during the longitudinal assessment.

7. Participant knowledge of the interviewer	What did the participants know about the researcher? e.g. personal goals, reasons for doing the research	Qualitative study participants and recruitment p. 14
8. Interviewer characteristics	What characteristics were reported about the inter viewer/facilitator? e.g. Bias, assumptions, reasons and interests in the research topic	RQ was specifically dedicated to participants' assessment and interviews. He did not participate in any other study activities.
**Domain 2: study design**		
Theoretical framework		
9. Methodological orientation and Theory	What methodological orientation was stated to underpin the study? e.g. grounded theory, discourse analysis, ethnography, phenomenology, content analysis	Qualitative analysis p. 5
Participant selection		
10. Sampling	How were participants selected? e.g. purposive, convenience, consecutive, snowball	Qualitative study participants and recruitment p. 14
11. Method of approach	How were participants approached? e.g. face-to-face, telephone, mail, email	Qualitative study participants and recruitment p. 14
12. Sample size	How many participants were in the study?	Nested qualitative study results p.14
13. Non-participation	How many people refused to participate or dropped out? Reasons?	Nested qualitative study results p.14
Setting		
14. Setting of data collection	Where was the data collected? e.g. home, clinic, workplace	Qualitative study participants and recruitment p. 14
15. Presence of non-participants	Was anyone else present besides the participants and researchers?	No
16. Description of sample	What are the important characteristics of the sample? e.g. demographic data, date	The demographic and clinical characteristics of the sample are individually presented in S6 Appendix.
Data collection		

17. Interview guide	Were questions, prompts, guides provided by the authors? Was it pilot tested?	Nested qualitative study p. 13, 14.<br>S3 Appendix.
18. Repeat interviews	Were repeat inter views carried out? If yes, how many?	Nested qualitative study p. 13, 14.
19. Audio/visual recording	Did the research use audio or visual recording to collect the data?	Nested qualitative study p. 13, 14.
20. Field notes	Were field notes made during and/or after the inter view or focus group?	Nested qualitative study p. 13, 14.
21. Duration	What was the duration of the inter views or focus group?	Nested qualitative study p. 13, 14.
22. Data saturation	Was data saturation discussed?	No, we decided to interview all the participants.
23. Transcripts returned	Were transcripts returned to participants for comment and/or correction?	No. The interviewer check the key points of the interview with each participant at the end of the interview.
**Domain 3: analysis and findings**		
Data analysis		
24. Number of data coders	How many data coders coded the data?	Qualitative analysis p. 15.
25. Description of the coding tree	Did authors provide a description of the coding tree?	Qualitative analysis p. 15.<br>S6 Appendix.
26. Derivation of themes	Were themes identified in advance or derived from the data?	Qualitative analysis p. 15.
27. Software	What software, if applicable, was used to manage the data?	We did not use any software.
28. Participant checking	Did participants provide feedback on the findings?	No.
Reporting		
29. Quotations presented	Were participant quotations presented to illustrate the themes/findings? Was each quotation identified? e.g. participant number	Nested qualitative study results p. 19 – 32.<br>S6 Appendix.
30. Data and findings consistent	Was there consistency between the data presented and the findings?	Yes
31. Clarity of major themes	Were major themes clearly presented in the findings?	Yes
32. Clarity of minor themes	Is there a description of diverse cases or discussion of minor themes?	Yes.
