# Supplementary material for: A resilience group training program for people with multiple sclerosis: Results of a pilot single-blind randomized controlled trial and nested qualitative study
Source: PLoS One. 2020 Apr 9;15(4):e0231380. doi: 10.1371/journal.pone.0231380 (PMC7145197; doi:10.1371/journal.pone.0231380)
Supplement: S3 Appendix — (PDF) [file pone.0231380.s003.pdf]

S3 Appendix - Study Protocol

Italian set up of the program "REsilience and Activity every DaY for MS", of outcomes, and pilot assessment of efficacy using a mixed methodology (READY-It-MS)

Study Design: Randomized controlled, pilot Study

Study population: Multiple Sclerosis patients

Promoting Centre and Study location: Foundation IRCCS Neurological Institute "Carlo Besta" Via Celoria 11, 20133 Milan. Italy.

Principal Investigator: Dr. Ambra Mara Giovannetti

Operative Units:

U.O. Neuroepidemiology (Head of the U.O.: Dr. Alessandra Solari)

UO Neurologia IV – Multiple Sclerosis Centre (Head of the U.O.: Dr. Renato Mantegazza).

August, 17, 2017

In case of adversity, people with low resilience may experience poor quality of life (QoL), emotional burden (i.e. depression, anxiety, stress), and interpersonal difficulties. Living with MS can be highly burdensome and it was demonstrated that persons with MS (PwMS) have poorer QoL than non-diseased controls and people with other chronic diseases; 50% reported high level of depressive symptoms and approximately 35% showed anxiety disorders [1,2]. Targeted intervention aimed at promoting personal resilience can alleviate adverse effects of stress of living with MS and sustain a better QoL [3]. The READY program has been demonstrated to effectively improve resilience. Pakenham et al. recently proposed a READY program specifically tailored for PwMS. It is of primary importance to: a) increase the scientific evidence on the efficacy of the READY program;b) to tailor it to Italian PwMS.

From coping to resilience

The Stress and Coping Model, developed by Lazarus and Folkman (1984), has played a central role in guiding research into the adjustment and coping processes involved with chronic illnesses, including MS [4].

In 2000, this framework has been expanded including emerging positive psychology movement and paying particular attention on resilience [5]. Resilience is the process of negotiating, managing and adapting to significant sources of stress or trauma. Individual resources and the environment may facilitate this capacity for adaptation [6], and psychological flexibility (the ability to defuse from difficult thoughts and accept difficult feelings while persisting in values-based action) resulted to play a key role in promoting it [7].

When facing adversity, people with low resilience have higher risk to experience poor QoL, emotional burden and interpersonal difficulties. Moreover they can adopt health compromising behaviors and experience somatic complains and poor physical health. Prolonged stress together with poor psychosocial functioning may negatively impact on physical health through different mechanisms, such as: hypertension and blood pressure reactivity to stress, pro-inflammatory cytokines and the development of metabolic syndrome [8].

A recent meta-analysis reported a modest but consistent benefit of resilience training programs in improving a number of mental health outcomes in adults living with chronic illnesses [3], and a study conducted in 2015 with PwMS supported the efficacy of interventions promoting individual resilience in this population [9].

MS experience and resilience

Patients' experience of MS is usually characterized by remissions, relapses, possible persistent disability and continuous progression. As a result, PwMS have often to deal with uncertainty about disease, loss of function, changes in life roles and a variety of symptoms [10]. Considering also that MS typically manifests in young adulthood, the impact of diagnosis is particularly distressing as it has the potential to significantly interfere with life goals [11]. For all these reasons, adjusting to MS can be highly demanding [12], and the disease can be a consistent source of stress. Moreover, evidence suggests an association between psychological stress and subsequent relapses in MS, with the occurrence of stressful life events purported to lead to a greater risk for relapses [13].

Given that, personal resilience can be seen as a potential internal resource for alleviating the adverse effects of stress and for sustaining good mental health through adversity [3], and PwMS may benefit from a targeted intervention aimed at promoting psychological flexibility and their resilience. However, empirical evidence regarding the benefits of applying resilience training to PwMS is limited.

ACT & the READY program

The Acceptance and Commitment Therapy (ACT) is an empirically based third generation cognitive behavioral approach specifically aimed at promoting psychological flexibility, the key ingredient of resilience, by targeting 6 positive psychological skills: acceptance, cognitive defusion, contact with the present moment, self as context, values, committed actions [14]. Each skill has been shown to be related to: better mental health, lower risk of disease, better health outcomes for those already diagnosed with illness, neurobiological resilience factors [15,16]. An ACT based intervention has been preliminary demonstrated to be effective in promoting better QoL and resilience in PwMS [17].

Pakenham et al. created a highly structured, ACT-based group intervention, READY, and developed a specific version for PwMS: “READY for MS”.

The READY program is designed to help people to be more resilient in their everyday life, learning how to manage the challenges and stress associated with work, relationships, health, daily hassles and life events.

READY is a group psychosocial resilience training program aimed to promote resilience by targeting the afore-mentioned 6 positive psychological skills. Sessions involve psycho-education, discussions, experiential exercises, and home assignments [18].

To date, some studies have been conducted to evaluate the effect of the READY program on the general population and on people with different health conditions [18,19]. In 2009 Burton and colleagues presented a methodological study preliminary to a randomized controlled trial (RCT) of the READY program for prevention of coronary heart disease [19]. In a subsequent pilot trial they gathered preliminary evidence that supported the feasibility of implementing the READY program in a workplace setting, and its ability in promoting well-being. They found significant improvement in various outcome measures, including mastery, positive emotions, personal growth, mindfulness, acceptance, stress, self-acceptance, valued living, autonomy, and plasma cholesterol levels [18].

The READY program was delivered also to persons with Diabetes (10 two-hour weekly group sessions): Participants reported greater resilience, stress management skills, mindfulness, acceptance, defusion, values driven living, with a mean satisfaction rating for the treatment of 4.7 on a 5-point rating scale [20].

Recently the same research group proposed a READY program specifically tailored for PwMS (see methodology session). Preliminary results showed a statistically significant decrease in the global distress dimension, particularly for depression and distress; with regards to ACT process, participants reported improvement in 3 dimensions: defusion, values and acceptance willingness [21].

To date, no studies have been performed in Italy, and participants' tools and materials are not available in Italian. Hence, it is of primary importance to: (a) increase the scientific evidence on the efficacy of the READY program; (b) and to tailor the program to Italian PwMS.

Methodological framework

We will follow the Medical Research Council (MRC) framework for developing and evaluating complex interventions, which has a phased approach, from a pre-clinical research phase to a final phase in which the intervention is introduced into the health service, leading to a theory-driven intervention: a "bottom up" development which guarantee to enter a phase III trial with an appropriate theory and pilot work [22].

Theoretical framework

ACT is based on Relational Framework Theory: this brings to the idea that the psychological events experienced by an individual involve the interaction between historically and situationally defined contexts [14]. For this reason, events cannot be viewed in isolation as their meaning depends upon the context in which they occur, and psychological and behavioral events do not cause one another directly; rather they influence each other within particular contexts.

According to ACT, psychopathology is conceptualized as being fundamentally the result of psychological inflexibility [14]. Essentially, the manner in which we relate to our inner mental experiences has the capacity to impede our ability to participate in valued living.

The goal of ACT is to create rich, full and meaningful lives whilst accepting the pain that inevitably ensues [23]. To achieve this, proponents of ACT endeavor to facilitate and foster the development of psychological flexibility by way of incorporating the six fundamental processes that make up the ACT Hexaflex into therapeutic frameworks for intervention [14] (see Appendix 1). The six core processes of ACT include acceptance, cognitive defusion, contact with the present moment, self as context, values, committed actions. In the last years, Pakenham and his group have developed a specific group training program (READY) to promote higher resilience, and they adapted it to MS ("READY for MS").

AIMS

The main goal of this study is to apply "READY for MS" in Italy.

Specific goals are:

1) to translate the READY for MS materials and manual, and linguistically validate into Italian the Drexel Defusion Scale (DDS) [24] and the "Comprehensive assessment Acceptance and Commitment Therapy processes (CompACT)" [25].

2) to preliminary evaluate the efficacy of "READY for MS" program in a single-blind, pilot randomized controlled trial (RCT) and nested qualitative study.

The RCT has the following aims:

Primary Aim: To verify that participants assigned to the "READY for MS" group show higher improvements in QoL, measured with the MHC of the 54-items MS Quality of Life inventory (MSQOL-54) compared to the control group (relaxation).

Secondary Aims:

- To verify that participants assigned to the "READY for MS" group show higher improvements in QoL, measured with the PHC of the 54-items MS Quality of Life inventory (MSQOL-54) compared to the control group (relaxation).
- To verify that participants assigned to the "READY for MS" group show higher improvements in mood (HADS; PSS), individualized quality of life (SEIQOL-DW), resilience (CDRISC-25), psychological flexibility (CompACT), and its protective factors: Acceptance (AAQ-II); Cognitive defusion (DDS); Contact with the present moment (MAAS); Values and committed actions (VLQ), compared to the control group (relaxation).
- To evaluate the correlation between the different outcome measures: health related quality of life (MSQoL-54), mood (HADS; PSS), individualized quality of life (SEIQOL-DW), resilience (CDRISC-25), psychological flexibility (CompACT) and its protective factors: Acceptance (AAQ-II); Cognitive defusion (DDS); Contact with the present moment (MAAS); Values and committed actions (VLQ), compared to the control group (relaxation).
- To verify that participants assigned to the "READY for MS" group show higher improvements in health related QoL (MSQoL-54), mood (HADS; PSS), individualized quality of life (SEIQOL-DW), resilience (CDRISC-25), psychological flexibility (CompACT) and its protective factors: Acceptance (AAQ-II); Cognitive defusion (DDS); Contact with the present moment (MAAS); Values and committed actions (VLQ) at each time point, compared to the control group (relaxation).

ENDPOINTS

Primary Endpoint: differences between changes in MHC scores at different time-points between READY and control groups.

Secondary Endpoints:

- differences between changes in PHC scores at different time-points between READY and control groups.
- differences between changes in HADS, PSS, SEIQoL-DW, CDRISC-25, CompACT, MAAS, VLQ, AAQII, DDS and scores at different time-points between READY and control groups.
- Correlation between patients reported outcome measures: MSQoL-54 (MHC, PHC), HADS, PSS, SEIQoL-DW, CDRISC-25, CompACT, MAAS, VLQ, AAQII, DDS.
- differences between changes in MSQoL-54 (MHC, PHC), HADS, PSS, SEIQoL-DW, CDRISC-25, CompACT, MAAS, VLQ, AAQII, DDS scores at each time-points between READY and control groups.

METHODOLOGY

Study design:

The project will last two years, and it is composed of two phases.

Phase 1: We will translate into Italian "READY for MS" Therapist and Participant manual and the DDS [24] and CompACT [25].
Translation and linguistic validation will follow accepted guidelines [26]. The main steps in this process are: 1. Forward translation. Two qualified translators will produce two independent translations. A panel consisting of the translators, a neurologist, a psychologist, and a lay person will review the forward translations and a consensus version will be arrived at.
2. Backward translation. The consensus translation generated in step 1 will be independently translated back into English by a third qualified translator, without access to the original DDS and CompACT and without consulting the other translators. At a meeting between those participating in step 1 and the backward translator, the backward translation will be compared with the original, and further refinements to the Italian version will be made; differences will be resolved by discussion.
3. Cognitive debriefing. The Italian DDS and CompACT will be administered to 5-10 PwMS of diverse education, age, and EDSS score. After questionnaire administration, participants will be interviewed by AMG (semi-structured interview) to check the conceptual equivalence and content validity of the DDS and CompACT translations.

Phase 2: A single blind RCT with a nested qualitative study will be performed (see Flowchart). Data will be collected via questionnaires immediately before (baseline visit, T0), after the intervention (T1, 7 weeks after baseline visits), the booster session (T2, 12 weeks after baseline visit) and at three month follow-up (T3, 24 weeks after baseline visit). At the end of follow-up, half of the participants assigned to "READY for MS" program will be individually interviewed to appraise their experience, also addressing program weaknesses and strengths. Additional process data will capture participants' attendance, homework completion, and facilitator perspectives on a weekly basis. The study will be performed at the MS centre of the Besta Institute after obtaining the Human Research Ethics Committee approval.

Participants' Eligibility

Subjects are eligible for recruitment if all the following criteria are satisfied: Diagnosis of MS [27]; Age>=18 years; Signed informed consent; The Connor-Davidson Resilience Scale (CDRISC-25) score <83, which indicates that the person could still improve his/her level of resilience); Able to attend the program group sessions (7 sessions, each lasting 2.5 hours); Fluent Italian speaker.
Subjects will be excluded from the study if one or more of the following criteria apply: Severe cognitive compromise (MMSE<19); Psychotherapy ongoing or in the preceding six months; Previous experience in meditation or other mind-body therapies; Major psychiatric disorders (including psychotic disorders or active substance abuse problems); Pregnancy; MS diagnosis for less than three months; One or more relapses in the last month.

Trial Procedures

Potential participants will be provide with a general overview of the study. Subsequently, one trained clinical psychologist (not involved with the treatment and blind to group allocation) will make an appointment with those patients who met the inclusion criteria and agreed to participate in the study. The psychologist will check all the eligibility criteria and perform the baseline evaluation

(T0). Information on all screened PwMS and reasons for exclusion will be recorded. After that the PwMS is assigned to "READY for MS" vs. "control" (see randomization below).
The interventions start within 2 weeks from the baseline assessment.

Withdraw
Participants will be free to withdraw from the study at any time, without giving reasons and with no risk of prejudicing future care. Study personnel will make every effort to obtain, and record, information about the drop out reasons.

Pre-study interview and informed consent (visit 0)
During the pre-study evaluation each potential participant receives full and adequate verbal and written information about the nature and purpose of the study. A written, signed informed consent is obtained, according to the Declaration of Helsinki and to the GCP Guidelines of the EU. The informed consent form will be kept on file by the study personnel and will be available for inspection by regulatory authorities or authorized persons.

Assessments
At baseline (T0), 8 weeks (T1), 12 weeks (T2) and 24 weeks after treatment beginning (T3) the PwMS completes the following PROMs (cited in order of administration): MSQOL-54, CDRISC-25, HADS, PSS, CompACT, MAAS, VLQ, AAQII, DDS. Further to questionnaire completion the examiner administers the SEIQoL-DW at T0, T2 and T3. The total assessment will last about 40 minutes in T1 and about one hour in all the other timepoints.

Randomization
Randomization will be provided by an independent randomization service at the Besta Neuroepidemiology Unit and accessed via a web-based system, using computer-based block randomization (2 factors: Expanded Disability Status Scale (EDSS) [28] score < 2.0 and >= 2.0; CDRISC-25 score < 50 and >=50). Patient will be allocated to two arms: "READY for MS" vs. relaxation program in a 1:1 ratio.
Confirmation e-mails will be sent to AMG.

Interventions
Each group will be composed of 8-10 participants, a total of 4 groups will be performed (2 "READY for MS", and 2 relaxation; within each arm, the two groups will be homogeneous assembled so that PwMS will be as much homogeneous as possible in terms of their EDSS score and CDRISC-25 score.

1) "READY for MS": it is an adult resilience training program based on ACT that comprises 7 modules of 2.5 hour weekly group sessions, with a 2.5 hour 'booster' session approximately 5 weeks after the final session of the intervention. The booster session starts with a mindfulness exercise, followed by a review of the contents covered across the READY program. Participants are encouraged to share their progress and experience of applying the strategies and techniques learned

through attending the READY program. All the sessions are guided by a facilitator (AMG, a trained psychotherapist). It incorporates a blend of psychoeducation and experiential exercises, combined with readings and homework exercises that participants are encouraged to practice between sessions (see Appendix 2).
2) Control treatment: it consists of a group relaxation program (7 one hour weekly group sessions, followed by a 'booster' session approximately after 5 weeks. This control program matches the study intervention in duration and schedule (but not in content), in order to control for the non-specific effect of the intervention. We decided to limit the duration to 1 hour, as 2.5 hours was judged too much for group relaxation.

Primary Outcome Measure
The MSQOL-54 is a health-related QoL measure that comprises the generic Short-Form 36-item (SF-36) [29], plus 18 MS-specific items [30,31]. The 54 items are organized into 12 multi-item and two single item subscales. As for SF-36, two composite scores (Physical Health Composite, PHC, and Mental Health Composite, MHC) are derived by combining scores of the relevant subscales. The MSQOL-54 has well documented validity in terms of content, constructs, reliability, discrimination, and responsiveness [31]. To limit multiple comparisons, we will primarily assess changes in PHC and MHC.

Secondary Outcomes
Mood
- The Hospital Anxiety and Depression Scale (HADS) is a well-validated measure that consists of two seven-item subscales to assess anxiety and depressive levels. Higher scores indicate higher level of depressive or anxiety symptoms. Unlike a number of other measures, the HADS excludes somatic symptoms of anxiety and depression, which may overlap with physical illness [32].
- The 10-item version of the Perceived Stress Scale (PSS) will be used to assess the extent to which life situations are appraised as stressful. Higher score indicated higher level of stress perceived [33].

Resilience
The Connor-Davidson Resilience Scale (CDRISC-25) is used to assess psychological resilience. It is composed of 25 items, each rated on a 5-point scale (0-4), with higher scores reflecting greater resilience.
The scale demonstrated good psychometric properties [34].

Psychological Flexibility
The CompACT scale consists of 23 items, each rated on a 0-6 Likert scale and grouped in three scales (openness to experience, behavioral awareness, and valued action). A total score is calculated as the sum of the three subscale scores (range 0-138, higher values indicating greater psychological flexibility). The CompACT demonstrated good internal consistency, and converged and diverged in theory-consistent ways with other measured variables: higher levels of psychological inflexibility were associated with higher levels of distress and lower levels of health and wellbeing [25].

Mindfulness

The Mindful Attention Awareness Scale (MAAS) is a 15-item scale aimed to assess a core characteristic of dispositional mindfulness across interpersonal cognitive, physical, emotional, and general domains. Items are rated on a 6-point Likert scale, and responses are then summed with higher scores indicating a greater presence of mindfulness. The MAAS has validity, internal reliability and sensitivity to change [35].

Values and Meaningful Action

The 20-item Valued Living Questionnaire (VLQ) measures the relative importance of certain life domains and the consistency of behaviours with the identified personal values. Respondents are asked to rate the 10 life domains on a 1–10 scale on level of importance (importance subscale) and how consistently they have lived in accord with those values in the past week (consistency subscale). Higher scores indicate greater importance and consistence. The VLQ displays good inter-item consistency, test-retest reliability, and construct validity [36].

Acceptance

The Acceptance and Action Questionnaire II (AAQ-II) is a 10-item self-report measure of acceptance and experiential avoidance. Items are rated using a 7-point Likert scale. High scores on the AAQ-II are reflective of greater experiential avoidance and immobility, while low scores reflect greater acceptance and action. It has been shown to have good internal reliability and convergent validity [37].

Defusion

The DDS measures psychological distance from a broad range of internal experiences incorporating both thoughts and feelings *(it is person's ability to see thoughts as what they are, not as what they say they are)*. Subjects are asked to read a definition of defusion prior to indicating the extent to which they would normally be in a state of defusion across ten different scenarios, using a 6-point Likert scale (higher scores indicating greater ability to defuse from distressing thoughts and feelings) [24].

Individualized QoL

It will be measured by the SEIQoL-DW, an interview-based instrument to assess the level of functioning in, and relative importance of, areas of life individually identified by the respondent. The evaluation is based on three steps: (a) to name the subject 5 most important QoL areas; (b), to rate the relative importance of each identified area, using a disk that can be rotated around a central point to form a type of pie chart (it displays a 0–100 scale); (c), to assign a satisfaction score to each of the five areas. The SEIQoL-DW index is obtained from the satisfaction and the weight of each elicited area, and can range from 0 (worst possible) to 100 (best possible) [38].

Clinical information and measures

The following information will be also provided by the PwMS neurologist at T0: EDSS score, MS course (relapsing remitting, primary progressive, secondary progressive), presence/type of co-pathologies, and ongoing treatment. At T1 and T2, the neurologist will update the EDSS score, treatment, and occurrence of new relapses.

Satisfaction with the READY

An ad hoc questionnaire has been built-up to explore the satisfaction with the READY program. It is composed of 3 sections: 1) Usefulness of the READY program in promoting the 6 protective factors of resilience (6 item). 2) Overall evaluation of the READY program (5 item, plus 8 open questions on their experience). 3) Satisfaction with the READY Personal Plan (5 Item, plus we ask the participants to rate the level of commitment with the READY Personal Plan, after each session).

DATA ANALISYS

Sample size calculation

We estimated a minimal sample size of 15 patients per arm in order to detect a large post-intervention effect size (d=0.64) on MSQOL-54 Mental Health Composite, with a power of 0.80 and a two-tailed a of 0.05. Assuming 20% dropout, the total sample size required is of 36 patients (see Appendix 3).

Our estimate was based on the large effect size on QoL (d=0.80) on the Profile of Health-related Quality of Life in Chronic Disorders scale) found on a RCT on group mindfulness [39], and on available data on MSQOL-54 Mental Health Composite [31,40].

Analysis

Continuous data will be described using frequency, mean, median, standard deviation, min and max. Longitudinal changes will be analyzed using linear mixed effects regression models with time visits as fixed effects [41]. Univariate and stratified analyses will be done on all clinically relevant covariables not included as a block factor in the randomization process (i.e. EDSS score at baseline, time from diagnosis).

Between-group comparisons will be done using either the two-sided unpaired t-test or the Wilcoxon two sided two-sample test for non-parametric data. Normality assumption will be tested with the Shapiro-Wilk normality test. Correlations will be computed using Spearman’s or Pearson’s coefficients depending on data distribution.

All tests will be two-tailed, and values of $p < 0.05$ will be considered significant. All data will be analyzed according to the intention-to-treat principle (ITT). A per protocol analysis will be also performed. Statistical analyses will be performed using Stata Statistical Software v12.0.

NESTED QUALITATIVE STUDY

A nested qualitative study will explore MS study arm patients’ experiences of treatment via semi-structured personal interviews. The objectives are to provide insight into the quantitative results, explore psychological processes of change, and factors related to program acceptance/adherence.

We will recruit approximately 5 participants per group (i.e. 50% of active group participants), sampled purposively to encompass a mix of gender, ages, education and disease severity. The interviewer will use an interview guide comprising open-ended questions and prompts designed to elicit participants' accounts of their experiences. Interviews will last a maximum of one hour, they will be audio-recorded and transcribed verbatim.

Participants will be fully informed of the aims and requirements of the study, and consent obtained. Interviews will be conducted within 3 months following treatment completion. The psychologist will first explain the purposes of the interview, then he/she will guide the interview.

Each semi-structured interview began with a general question about the experience made during the READY program, "Did the READY program impact on in your resilience?"

Psychologist used, if necessary, additional prompts to facilitate the elaboration of narratives and to favor the in depth description of the lived experience (i.e. Did you observe any changes in your thinking, feelings, social relations, being, or behaviour, as a result of the READY program?" "What are the most helpful skills you learnt from the READY program?" "What you would like to change of the READY program?" "Which are the strength and the weakness of the READY program?" "Has the READY program impacted on how you feel, think about, or manage your MS? In which way?")

Thematic analysis will be used to code the data and to identify themes that capture key concepts and processes; it will begin on completion of the first few interviews and proceed iteratively, thus allowing early insights to be explored more fully in later interviews and interview guide to be modified if necessary [42]. Analysis is inductive and involves line-by-line coding with codes and categories derive from narratives. A two-step coding scheme will be applied. The first level codes come from sentences used directly by participants. This allows critical and analytical examination of the data, generation of new ideas and indications to further data collection. A second step will be used to aggregate data and to further refine the emerging codes and categories.

PROJECT DURATION

The project lasts 24 months. For details see the GANTT.

EXPECTED RESULTS AND IMPACT

This study will provide the following deliverables: the "READY for MS" program (materials and manual) for use in Italy; Italian version of the DDS [24] and the CompACT [25] for use in research and clinical practice.

In addition, we will produce evidence on a treatment to promote resilience in PwMS: for the first time, the READY for MS program will be compared with a control treatment (group relaxation) with the purpose to evaluate its specific effect. We expect that, by empowering participant inner resources, "READY for MS" can promote a personal growth that may help PwMS to prevent or overcome difficulties in adjustment to MS, and to live a full and rich life. The "READY for MS" program is brief and highly structured, which ease its affordability.

Importantly, all program activities can be performed by PwMS independently from their level of physical functioning, which makes it inclusive and accessible.

Ethics and Administrative Considerations

Ethical Considerations

This clinical study was designed and shall be implemented and reported in accordance with the ICH Harmonized Guidelines for Good Clinical Practice, with applicable local regulations, and with the ethical principles laid down in the Declaration of Helsinki.

Ethics Committee Approval

The protocol, Subject Information Sheet, Informed Consent Form and any advertisement for the recruitment of subjects must be reviewed and approved by an appropriately constituted Ethics Committee (EC), as required in chapter 3 of the ICH E6 Guideline. Written EC approval must be obtained by the Sponsor prior to shipment of study agent or subject enrolment.

Subject Information and Informed Consent

Eligible subjects may only be included in the study after providing written (witnessed, where required by law or regulation), EC-approved informed consent, or, if incapable of doing so, after such consent has been provided by a legally acceptable representative of the subject.
In cases where the subject’s representative gives consent, the subject should be informed about the study to the extent possible given his/her understanding. If the subject is capable of doing so, he/she should indicate assent by personally signing and dating the written informed consent document or a separate assent form. Informed consent must be obtained before conducting any study-specific procedures (i.e. all of the procedures described in the protocol).
The process of obtaining informed consent should be documented in the subject source documents. No study procedure can be performed before the written informed consent has been provided.

Confidentiality

Patient medical information obtained by this study is confidential and may only be disclosed to third parties as permitted by the Informed Consent Form (or separate authorization for use and disclosure of personal health information) signed by the patient, unless permitted or required by law. Medical information may be given to a patient‘s personal physician or other appropriate medical personnel responsible for the patient‘s welfare, for treatment purposes.
Data generated by this study must be available for inspection upon request by representatives of the national and local health authorities, monitors, representatives, and collaborators, and the IRB/EC for each study site, as appropriate.

Protocol Amendments

Any protocol amendments will be prepared by the Principal Investigator. Protocol amendments will be submitted to the EC and to regulatory authorities in accordance with local regulatory requirements.
Approval must be obtained from the EC and regulatory authorities (as locally required) before implementation of any changes, except for changes necessary to eliminate an immediate hazard to

patients or changes that involve logistical or administrative aspects only (e.g. change in monitor or contact information).

Study Management and Monitoring

Protocol deviation

A deviation to the protocol is defined as an event in which the investigator cannot conduct the study according to the protocol.

Source Documents

Source Documents (SD) are defined as original documents, data and records. These may include hospital records, medical records / outpatient data / information laboratory, data recorded from automated instruments, etc. Investigators should conserve all the source documents as required in the study protocol for at least 2 years after the end of the study.

Archiving of Records

The investigator is responsible for recording and storing the essential documents of the study, according to what / and for the time required by law and by GCP.

The Investigator must maintain adequate and accurate records to enable the conduct of the study to be fully documented, including but not limited to the protocol, protocol amendments, Informed Consent Forms, and documentation of EC and governmental approval. In addition, at the end of the study, the Investigator will receive the patient data, which includes an audit trail containing a complete record of all changes to data.

Auditing on Site

In the event that the investigator will be contacted by the Competent Authority in relation to this study, he or she will be required to immediately notify the Sponsor.

The investigator must be available to respond to requests and queries by inspectors during the audit process. The investigator must provide the Sponsor copies of all correspondence that may affect the revision of the current study.

Use and Publication of Study Results

The results of the study may be presented during scientific symposia or published in a scientific journal only after review and written approval by the involved parties in full respect of the privacy of the participating subjects.

Insurance Policy

The Neurological Institute Carlo Besta IRCCS Foundation has an adequate insurance policy to cover possible damages emerging from this RCT, pilot study.

References

1. Siegert RJ, Abernethy DA. Depression in multiple sclerosis: a review. J Neurol Neurosurg Psychiatry.2005;76:469-75
2. Korostil M, Feinstein A. Anxiety disorders and their clinical correlates in multiple sclerosis patients.Mult Scler.2007;13:67-72
3. Leppin AL et al. The efficacy of resiliency training programs: a systematic review and meta analysis of randomized trials. PLoS One.2014;9:e111420
4. Lazarus RS, Folkman S(1984). Stress, appraisal, and coping. NY: Springer
5. Seligman MEP, Csikszentmihalyi, M. Positive psychology: An introduction. Am Psychol.2000;55:5-14
6. Windle G et al. A methodological review of resilience measurement scales. Health Qual Life Outcomes.2011;9:8
7. Kashdan TB, Rottenberg J. Psychological flexibility as a fundamental aspect of health. Clin Psychol Rev.2010;30:865-78
8. Strike PC, Steptoe A. Psychosocial factors in the development of coronary artery disease. Prog Cardiovasc Dis.200446:337-47
9. Black R, Dorstyn D. A biopsychosocial model of resilience for multiple sclerosis. J Health Psychol.2015;20:1434-44
10. Dennison L et al. A review of psychological correlates of adjustment in patients with multiple sclerosis. Clin Psychol Rev.2009;29:141-53
11. Giordano A et al. Anxiety and depression in multiple sclerosis patients around diagnosis. J Neurol Sci.2011;307:86-91
12. Aikens JE et al. A replicated prospective investigation of life stress, coping, and depressive symptoms in multiple sclerosis. J Behav Med.1997;20:433-45
13. Mohr DC et al. Association between stressful life events and exacerbation in multiple sclerosis: a metaanalysis. BMJ.2004;328:731
14. Hayes SC et al. Acceptance and commitment therapy: Model, processes and outcomes. Behav Res Ther.2006,44:1-25
15. Southwick SM, Vythilingam M, Charney DS. The psychobiology of depression and resilience to stress: implications for prevention and treatment. Annu Rev Clin Psychol.2005;1:255-91
16. Ryff CD, Singer B (2003). The role of emotion on pathways to positive health. In Davidson RJ, Scherer KR, Goldsmith HH (Eds.), Handbook of affective sciences. New York: Oxford University Press
17. Sheppard SC et al. A Novel Application of Acceptance and Commitment Therapy for Psychosocial Problems Associated with Multiple Sclerosis. Int J MS Care.2010,12,4,200-6.
18. Burton NW et al. Feasibility and effectiveness of psychosocial resilience training: a pilot study of the READY program. Psychol Health Med.2010 May;15:266-77
19. Burton NW et al. Evaluating the effectiveness of psychosocial resilience training for heart health, and the added value of promoting physical activity: a cluster randomized trial of the READY program. BMC Public Health.2009;9:427

20. Ryan A, 2014. ACT and be READY: Evaluation of an ACT-based resilience training program delivered to people with diabetes. Doctoral thesis
21 Mawdsley, M. (2015). ACT and be READY: Evaluation of an ACT-Based Intervention for People Living with Multiple Sclerosis. Doctoral dissertation available from the University of Queensland.
22. Craig P et al. Developing and evaluating complex interventions: the new Medical Research Council guidance. BMJ.2008;337:a1655
23. Harris R (2009). ACT Made Simple: An Easy-To-Read Primer on Acceptance and Commitment Therapy.Oakland, CA: New Harbinger
24. Forman EM et al. The Drexel defusion scale: A new measure of experiential distancing. J Contextual Behav Sci.2012;1:55–6525
25. Francis AW, Dawson DL, Golijani-Moghaddam N. The development and validation of the Comprehensive assessment of Acceptance and Commitment Therapy processes (CompACT).J Contextual Behav Sci. 5(2016)134–145.
26. Wild D et al. ISPOR principles of good practice: the cross-cultural adaptation process for patient reported. Value Health.2005;8:94–104
27. Polman CH et al. Diagnostic criteria for multiple sclerosis: 2010 revisions to the McDonald criteria. Ann Neurol.2011;69:292–302
28. Kurtzke JF. Rating neurologic impairment in multiple sclerosis: an expanded disability status scale (EDSS). Neurology.1983;33:1444-52
29. Vickrey BG et al. A health-related quality of life measure for multiple sclerosis. Qual Life Res.1995;4:187–206
30. Solari A et al. Validation of Italian multiple sclerosis quality of life 54 questionnaire. J Neurol Neurosurg Psychiatry.1999;67:158–62
31. Giordano A et al. Responsiveness of patient reported outcome measures in multiple sclerosis relapses: the REMS study. J Neurol Neurosurg Psychiatry.2009;80:1023–28
32. Costantini M et al. Detecting psychological distress in cancer patients: validity of the Italian version of the Hospital Anxiety and Depression Scale. Support Care Cancer.1999;7):121-7
33. Cohen S, Kamarck T, Mermelstein R. A global measure of perceived stress. J Health Soc Behav.1983;24:386-396
34. Connor KM, Davidson JRT. Development of a new resilience scale: The Connor-Davidson resilience scale (CD-RISC). Depress Anxiety.2003;18:76-82
35. Brown KW, Ryan RM. The benefits of being present: mindfulness and its role in psychological well-being. J Pers Soc Psychol.2003;84:822-48
36. Wilson KG et al. The Valued Living Questionnaire: Defining and Measuring Valued Action within a Behavioral Framework. Psychol Rec,2010;60,249–72
37. Hayes SC et al. Measuring experiential avoidance: A preliminary test of a working model. Psychol Rec.2004;54,553-78
38. Hickey AM et al. A new short form individual quality of life measure (SEIQoL-DW): application in a cohort of individuals with HIV/AIDS. BMJ.1996;313:29–33
39. Grossman P et al. MS quality of life, depression, and fatigue improve after mindfulness training: a randomized trial. Neurology.2010;75:1141–9

40. De Giglio L et al. A low-cost cognitive rehabilitation with a commercial video game improves sustained attention and executive functions in multiple sclerosis: a pilot study. Neurorehabil Neural Repair.2015;29:453-61

41. Diggle P et al. Analysis of longitudinal data. 2nd ed. Oxford University Press;2002

42. Braun V, Clarke V. Using thematic analysis in psychology. Qual Res Psychol. 2006;3:77-101

APPENDIX 1: ACT Hexaflex

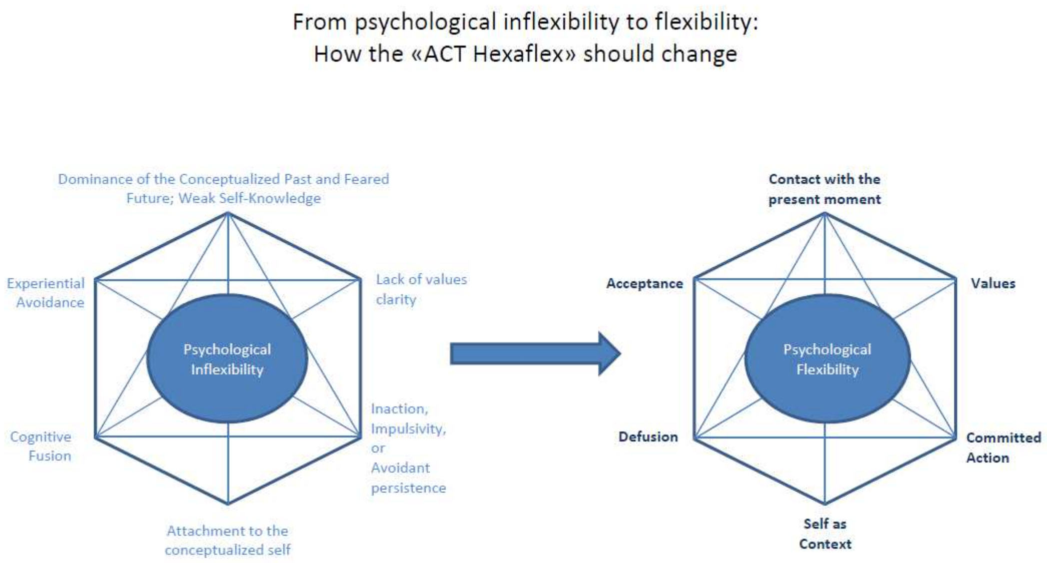

APPENDIX 2: READY for MS Sessions

(This material is provided by Prof. Kenneth Pakenham, Faculty of Psychology, University of Queensland)

Session 1: Introduction to the READY program
The introduction session aimed to: (1) build rapport; (2) outline the structure, purpose, and theoretical orientation of the READY Program and the READY personal plan; and (3) educate around protective factors for resilience and early warning signs of low resilience.

Session 1 introduced participants and facilitator and established group ground rules (e.g., participants can withdraw at any time, all information is confidential).
The READY program was outlined and each session discussed in terms of aims, main strategies used, and desired outcomes. The program content entailed psychoeducation on resilience, examined the READY model of resilience, and outlined the protective factors for resilience (i.e., cognitive flexibility, meaning, social connectedness, coping strategies, and acceptance). Participants were encouraged to reflect on general and personal signs of low resilience and note these in their READY personal plan. The content of Session 1 was then reviewed prior to conclusion of the session.

Session 2: Mindfulness
The aims of the mindfulness session were to: (1) review the previous session and READY personal plan activities; (2) review the READY resilience model; (3) introduce mindfulness; and (4) practise mindfulness exercises. Session 2 began with a review of Session 1 to reinforce concepts of resilience and protective factors. Psychoeducation was conducted on mindfulness and its importance in resilience, and compared to the unhelpful role of 'mindlessness' in daily stress. A variety of mindfulness exercises were practised including mindfulness of eating a sultana, mindfulness of sound and sight, mindfulness of breathing, and mindfulness of physical sensations. Participants were encouraged to share their experiences within the group setting following each exercise. Prior to the conclusion of Session 2, participants were given formal and informal mindfulness exercises to practise between sessions and incorporate into their READY personal plan.

Session 3: Defusion I
The aims of the first defusion session were to: (1) review the previous session, READY personal plan activities, and READY resilience model; (2) educate participants on fusion and defusion from thoughts; (3) teach participants to identify unhelpful thoughts; and (4) practise defusion strategies. Session 3 began with a mindfulness exercise and a review of the content delivered in Session 2, including participant progress with the mindfulness strategies delivered during that session. Education was conducted on the differences between thought fusion and defusion, and a variety of defusion strategies were delivered with participants encouraged to practice these during the session and review their experience within the group. Participants were asked to identify unhelpful thoughts, practise formal and real-time defusion, and keep a record of their practise between sessions. Prior to the conclusion of Session 3, participants were given formal and informal defusion exercises to practise between sessions and incorporate into their READY personal plan.

Session 4: Defusion II and the Observer Self

The aim of the second defusion session included the following: (1) review the previous session, READY personal plan activities, and READY resilience model; (2) trouble shoot defusion strategies learnt in Session 3; (3) practise additional defusion strategies; (4) educate participants on the "observer' self"; and (5) help participants identify unhelpful stories about the self. Session 4 began with a mindfulness exercise and a review of the content delivered in Session 3 to explore the progress that participants had made with defusion over the previous week. The session focused on troubleshooting any difficulties that participants identified in practicing the defusion strategies already delivered prior to the introduction of additional defusion strategies. Participants were encouraged to practise the additional defusion techniques during the session, and then review their experience with the group. Psychoeducation was delivered regarding the concept of the 'Observer Self' in contrast to the 'Conceptualised Self'.
Participants reflected upon the thoughts, images, and memories that substantiate their conceptualised self (i.e., personal stories), explored the impact of changing them, and considered the potential of adopting/ modifying new stories of themselves in the context of living with a diagnosis of MS. Prior to the conclusion of Session 4, participants were encouraged to utilise their READY personal plan to reflect further on their 'stories' and continue to practice the defusion strategies delivered during the session.

Session 5: Acceptance

The aims of the acceptance session were to: (1) review the previous session and READY personal plan activities; (2) review the READY resilience model; (3) educate participants on emotions and emotion management strategies; (4) educate participants on the concept of acceptance; and (5) practise acceptance strategies. Session 5 began with a review of the content delivered during Session 4 and a mindfulness exercise. Education and discussion were conducted in regard to emotion, experiential avoidance, and behavioural and cognitive methods for avoidance. Acceptance (allowing thoughts to exist and acknowledging the discomfort without struggle) was presented as an alternative strategy to manage uncomfortable emotions. Various experiential acceptance exercises were delivered during the session (e.g., the "Stop; Notice the unwanted feeling, thought, bodily sensation, memory, or image; Let go of the struggle; Make space for it" strategy) were practised. As with previous sessions, participants were encouraged to share their experiences among the group following experiential practice of each exercise. Prior to the conclusion of Session 5, participants were encouraged to reflect on their emotional learning and practise acceptance strategies daily, recording their experiences in their READY personal plan.

Session 6: Values and Meaningful Action

The aims of the values and committed action session were to: (1) review the previous session, READY personal plan activities, and READY resilience model; (2) educate participants on values; (3) assist participants to develop a value statement; and (4) assist participants to develop meaningful action consistent with their values; (5) educate participants on social connectedness and resilience, types of useful social support responses, and identify barriers to participating in social support; and (6) explore self-care strategies to promote resilience. Session 6 began with a mindfulness exercise and a review of the content delivered in Session 5, including the progress participants had made in their practice of the acceptance strategies over the previous week. The importance of personal values and meaningful action was discussed, including the difference between values, goals, and feelings. Participants were encouraged to examine their own personal values and ideal behaviours across various life domains (i.e., family, intimate relationships, and health), develop a values statement, review the consistency between their actions and values, and develop a new, values-consistent action. Prior to the conclusion of Session 6, participants were encouraged to implement

the meaningful action they identified during the session over the coming week, as well as identify another value and meaningful action and incorporate this into their READY personal plan.

Session 7: Finale and Future Planning

The aim of the final session was to: (1) review the previous session, READY personal plan activities, and READY resilience model; (2) understand the links between life domains, protective factors, and strategies to build resilience; (3) identify and demonstrate strategies to promote meaning, social support and connectedness, and relaxation; (4) identify resilient and non-resilient traits in relationships with others, meaning, and doing; (5) identify potential barriers to implementing resilience strategies, and ways to resolve these; and (6) refine a personal plan to identify and address early warning signs of low resilience. Session 7 began with a mindfulness exercise and a review of the content delivered in Session 6. Discussion was held around participant progress with regard to their identification of personal values and implementation of meaningful action. The session content focused on reviewing all important aspects of the program, synthesising key learnings, and ensuring participants had an applied understanding of the skills delivered throughout the intervention. The main areas reviewed included the characteristics of resilience and non-resilience, protective factors for resilience, meaning, social connectedness, coping strategies, cognitive flexibility, and acceptance. Resilience building strategies were reviewed and mapped onto the key protective factors. The group ended with the facilitator thanking all participants for their engagement, and asking them to each discuss one or two things they were going to take away from the program.

Booster session (approximately 5 weeks following Session 7)**:**

The booster session commenced with a mindfulness exercise and reviewed the content covered across the READY program. Participants were encouraged to share their progress and experience of applying the strategies and techniques learned through attending the READY program.

APPENDIX 3: Sample size calculation

Estimated sample size for two samples with repeated measures
Assumptions:
alpha = 0.0500 (two-sided)
power = 0.8000
m1 = 56
m2 = 70
sd1 = 22
sd2 = 22
n2/n1 = 1.00
number of follow-up measurements = 3
correlation between follow-up measurements = 0.750
number of baseline measurements = 1
correlation between baseline & follow-up = 0.750

```
Method: CHANGE
 relative efficiency =    3.000
    adjustment to sd =    0.577
        adjusted sd1 =   12.702
        adjusted sd2 =   12.702
```

```
 Estimated required sample sizes:
                  n1 =       15
                  n2 =       15
```
