# Supplementary material for: A resilience group training program for people with multiple sclerosis: Results of a pilot single-blind randomized controlled trial and nested qualitative study
Source: PLoS One. 2020 Apr 9;15(4):e0231380. doi: 10.1371/journal.pone.0231380 (PMC7145197; doi:10.1371/journal.pone.0231380)
Supplement: S4 Appendix — (PDF) [file pone.0231380.s004.pdf]

S4 Appendix – Interview guide

Interview Guide A

Dear [*participant's name*]
We are interested in better understanding your experience with the READY for MS. We would really appreciate if you will be willing to answer to some open ended questions.

1. **Could you please tell us about your experience with the READY program?**
   a. What are the most helpful skills you learnt from the READY program?
   b. What would you like to change of the READY program?
      i. Do you think that frequency and duration of the sessions were adequate?
      ii. Do you think that materials (Manual and audio recordings) were useful and adequate?
      iii. What do you think about the home activities (READY personal plan)?
   c. Which are the strength and the weakness of the READY program?

2. **Did you observe any changes in your thinking, feelings, social relations, being, or behavior, because of the READY program?**
   a. When did change begin?
   b. How was the trend of this change?
   c. Did it last?

3. **Do you think that your participation to the READY program changed your relation with MS? If yes, how?**

4. **Do you have any other comments?**

Interview Guide B

Dear [*participant's name*]
You participated in the relaxation treatment first, and after follow-up completion, you took also part in the READY training program.
We are interested in better understanding your experience with both the relaxation and READY for MS programs. We would really appreciate if you will be willing to answer to some open ended questions.

1. **Could you please tell us about your experience regarding both these interventions?**

2. **Could you please describe your experience with the relaxation program?**
   a. What are the most helpful skills you learnt from the relaxation program?
   b. What would you like to change of the relaxation program?
      i. Do you think that frequency and duration of the sessions were adequate?
      ii. Do you think that materials (training manual and audio recordings) were useful and adequate?
      iii. What do you think about the home activities?
   c. Which are the strength and the weakness of the relaxation program?

3. **Did you observe any changes in your thinking, feelings, social relations, being, or behavior, because of the relaxation program?**
    a. When did change begin?
    b. How was the trend of this change?
    c. Did it last?

4. **Could you please describe your experience with the READY program?**
    a. What are the most helpful skills you learnt from the READY program?
    b. What would you like to change of the READY program?
        i. Do you think that frequency and duration of the sessions were adequate?
        ii. Do you think that materials (Manual and audio recordings) were useful and adequate?
        iii. What do you think about the home activities (READY personal plan)?
    c. Which are the strength and the weakness of the READY program?

5. **Did you observe any changes in your thinking, feelings, social relations, being, or behavior, because of the READY program?**
    a. When did change begin?
    b. How was the trend of this change?
    c. Did it last?

6. **Did you notice any differences between relaxation and READY?**

7. **Which program did you prefer? Why?**

8. **Do you think that your participation to this study changed your relation with MS? If yes, how?**
    - What do you think was the reason of this change? (i.e. the group setting, meeting other people with MS, specific features of the program?)

9. **Do you have any other comments?**
