# Supplementary material for: A resilience group training program for people with multiple sclerosis: Results of a pilot single-blind randomized controlled trial and nested qualitative study
Source: PLoS One. 2020 Apr 9;15(4):e0231380. doi: 10.1371/journal.pone.0231380 (PMC7145197; doi:10.1371/journal.pone.0231380)
Supplement: S5 Appendix — (PDF) [file pone.0231380.s005.pdf]

S5 Appendix - Sample size calculation

Estimated sample size for two samples with repeated measures
Assumptions:
alpha = 0.0500 (two-sided)
power = 0.8000
m1 = 56
m2 = 70
sd1 = 22
sd2 = 22
n2/n1 = 1.00
number of follow-up measurements = 3
correlation between follow-up measurements = 0.750
number of baseline measurements = 1
correlation between baseline & follow-up = 0.750

Method: CHANGE
relative efficiency = 3.000
adjustment to sd = 0.577
adjusted sd1 = 12.702
adjusted sd2 = 12.702

Estimated required sample sizes:
n1 = 15
n2 = 15
