# Supplementary material for: A resilience group training program for people with multiple sclerosis: Results of a pilot single-blind randomized controlled trial and nested qualitative study
Source: PLoS One. 2020 Apr 9;15(4):e0231380. doi: 10.1371/journal.pone.0231380 (PMC7145197; doi:10.1371/journal.pone.0231380)
Supplement: S6 Appendix — (PDF) [file pone.0231380.s006.pdf]

S6 Appendix - Graphic plots for repeated measure analysis

MSQOL – 54 MHC

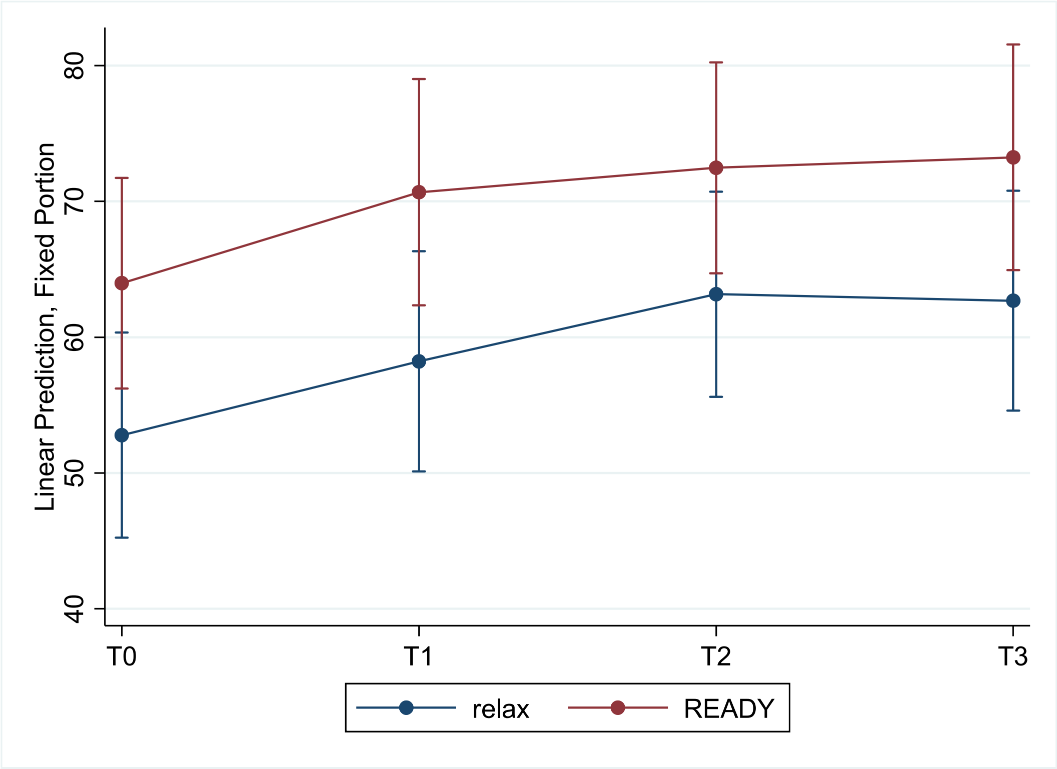

MSQOL – 54 PHC

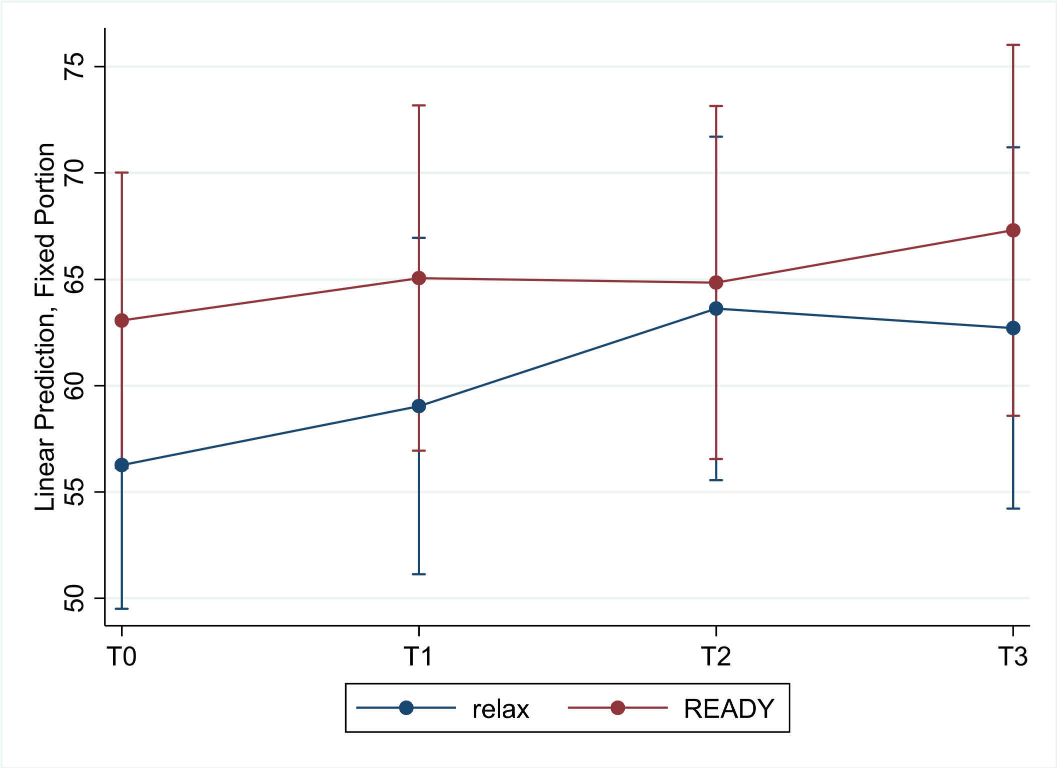

PSS

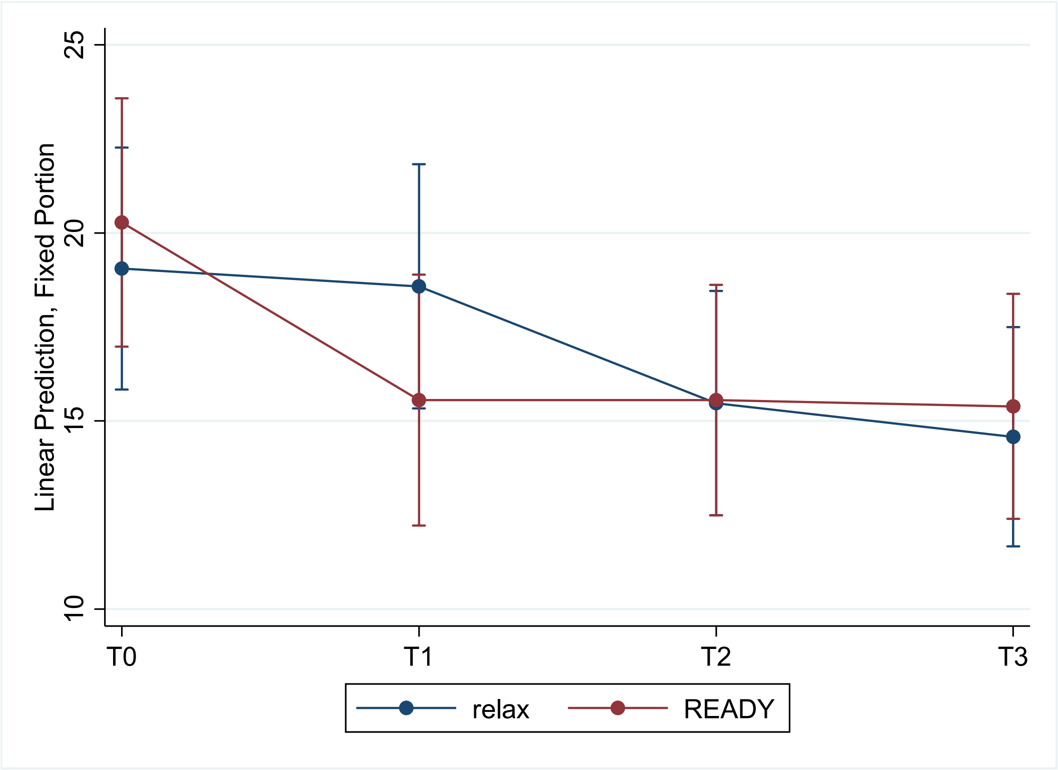

Linear Prediction, Fixed Portion
25
20
15
10
T0
T1
T2
T3
relax
READY

CD-RISC

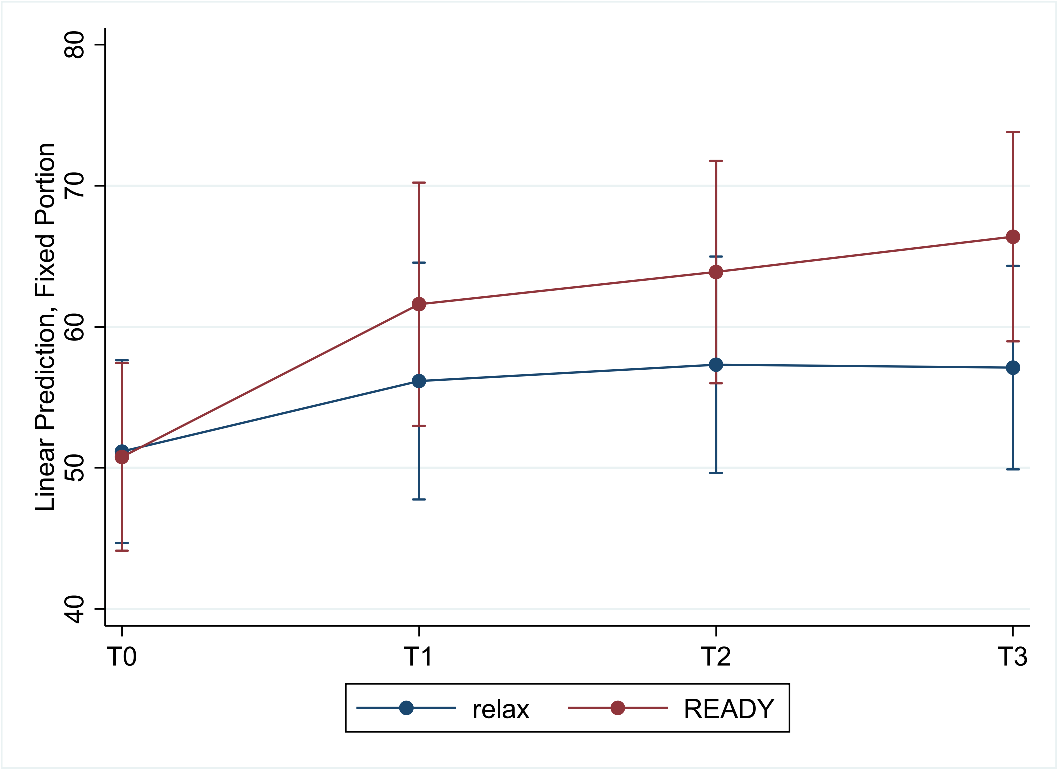

Linear Prediction, Fixed Portion
80
70
60
50
40
T0
T1
T2
T3
relax
READY

HADS-Anxiety

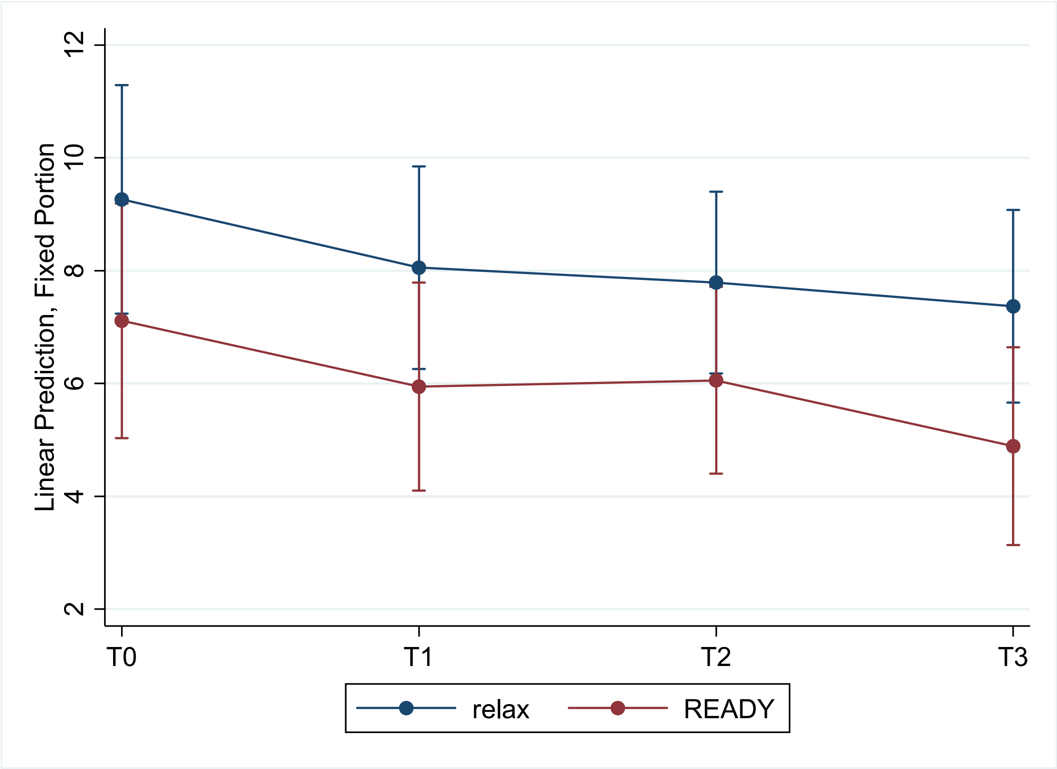

HADS-Depression

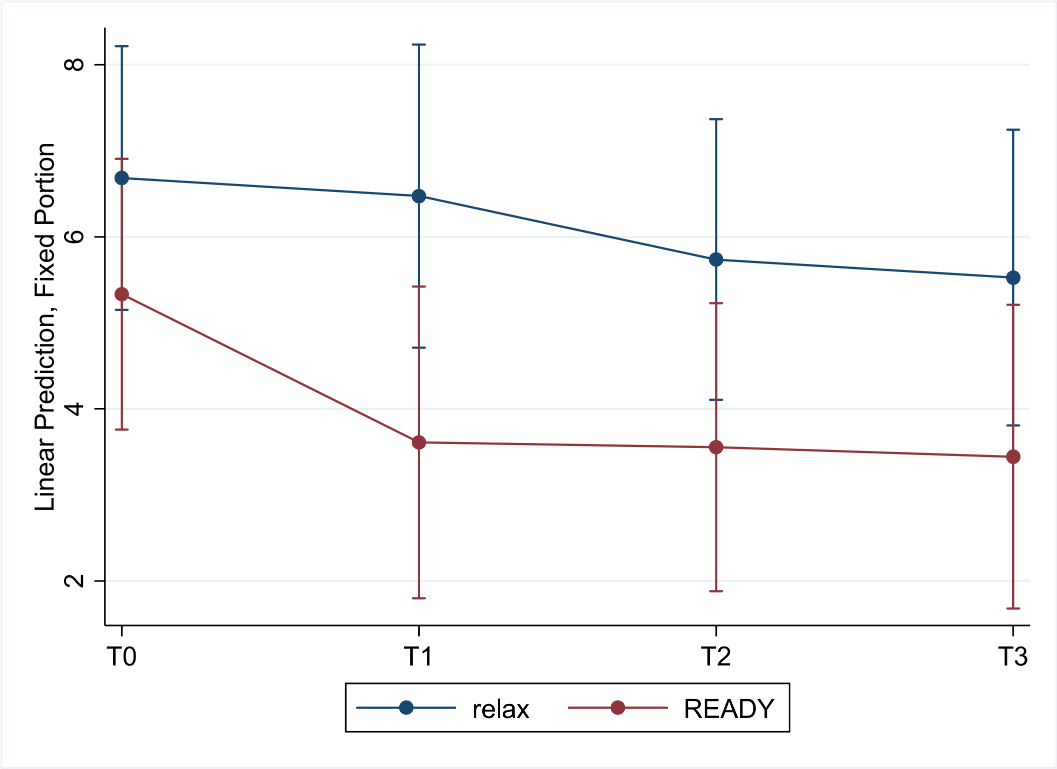

CompACT Total score

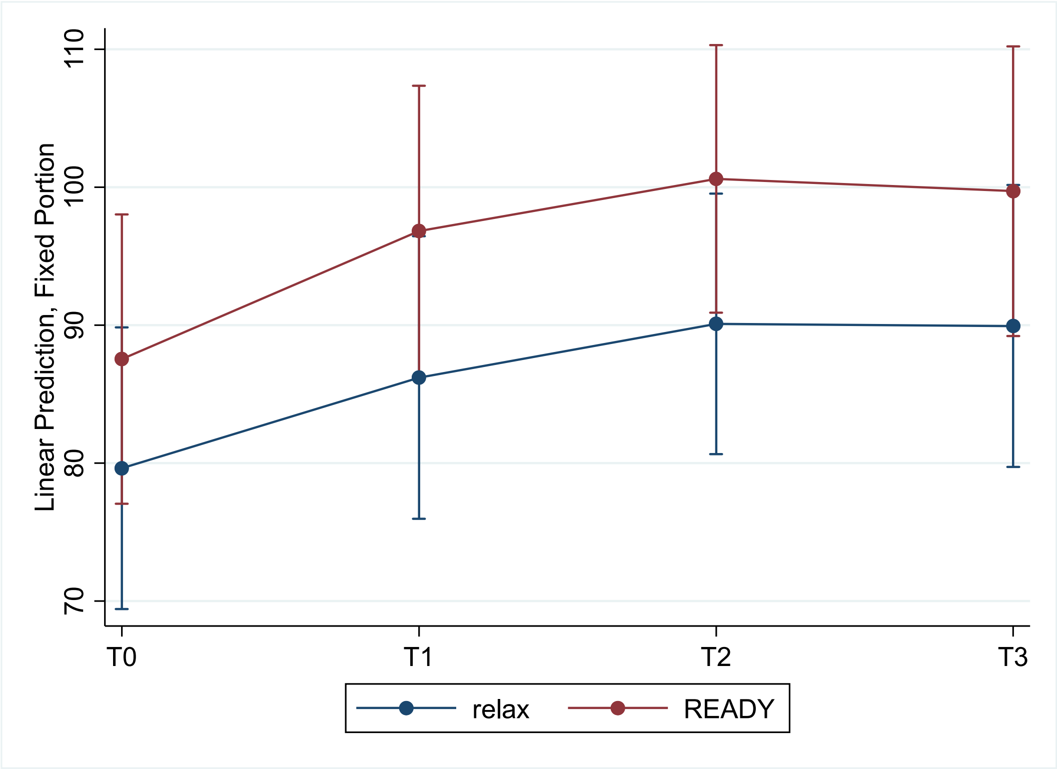

CompACT - Openness to the experience

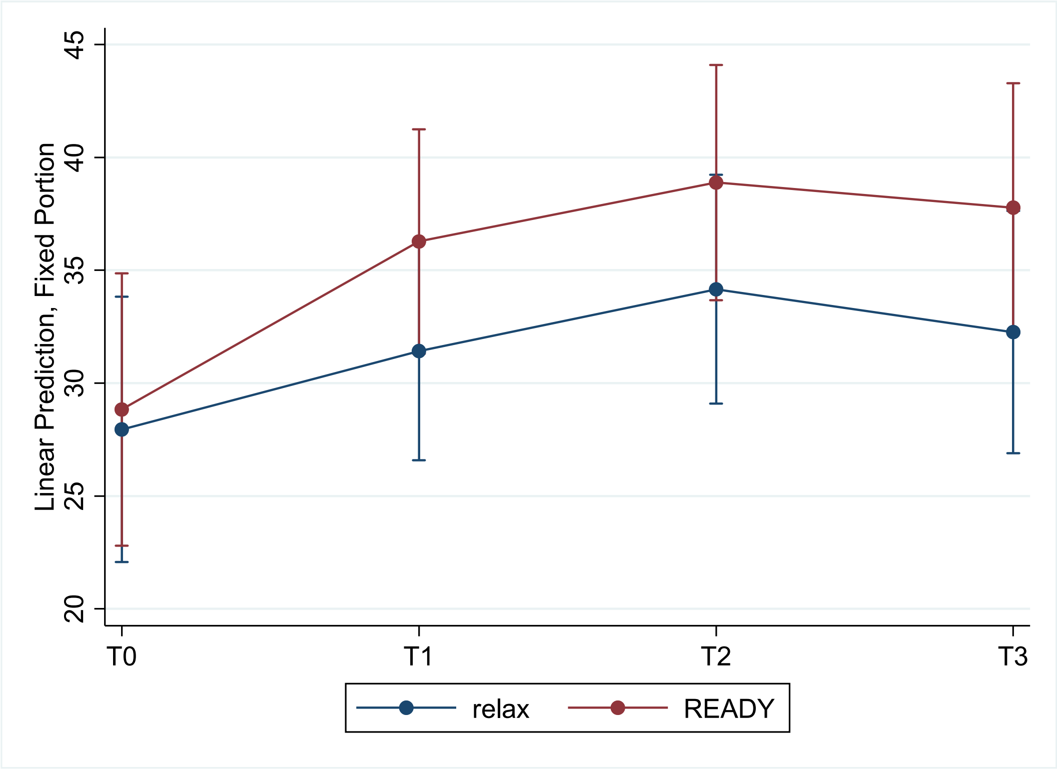

CompACT- Behavioral awareness

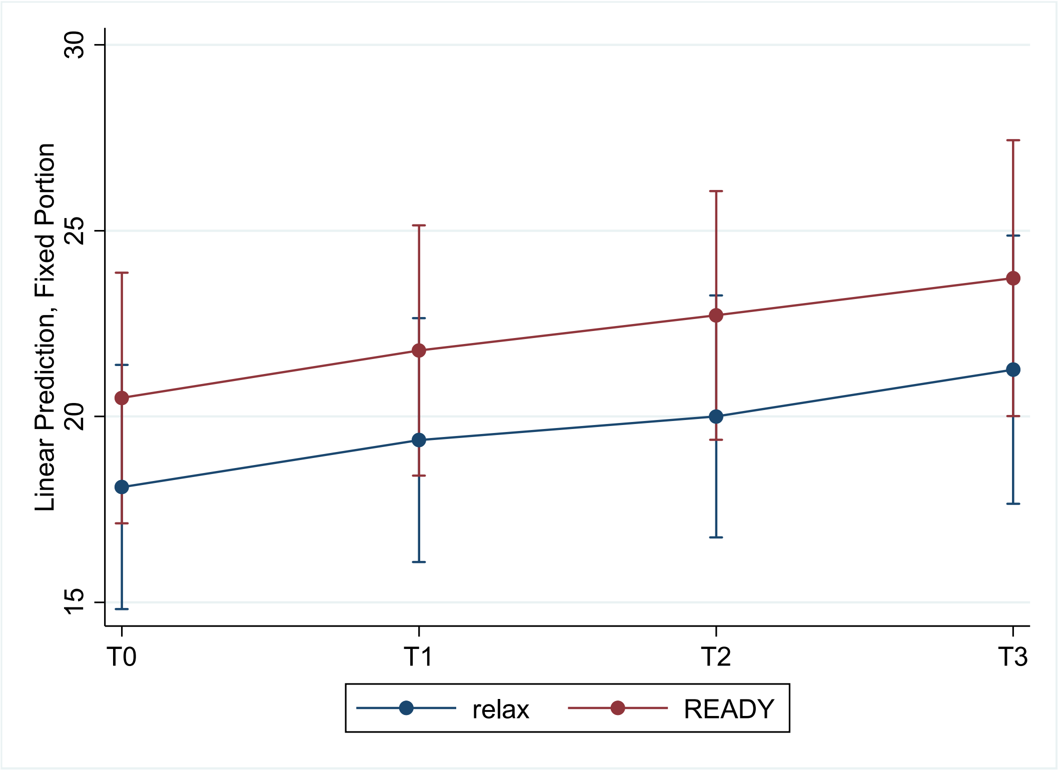

CompACT – Valued action

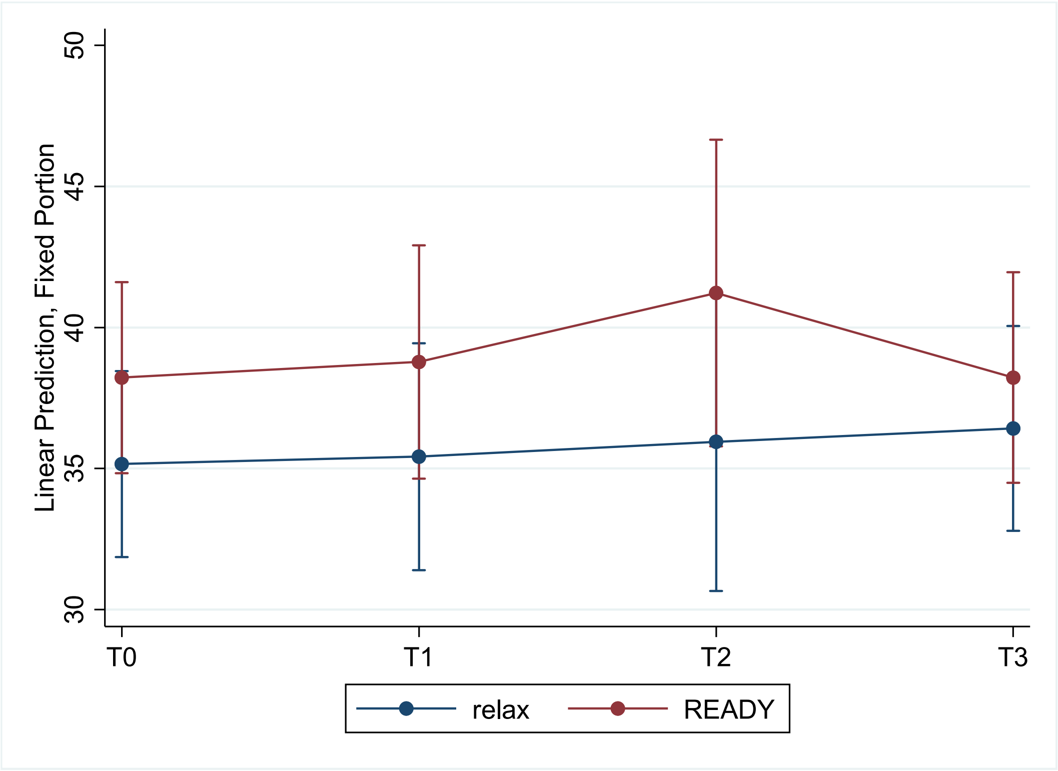

MAAS

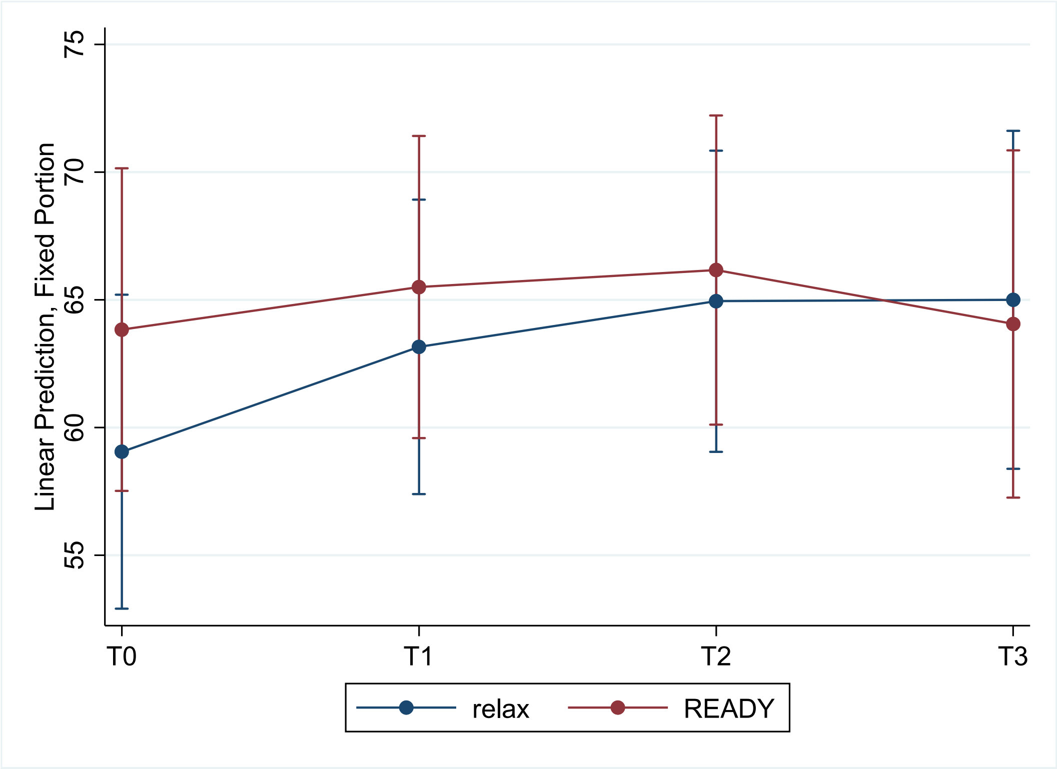

Linear Prediction, Fixed Portion
75
70
65
60
55
T0
T1
T2
T3
relax
READY

AAQ II

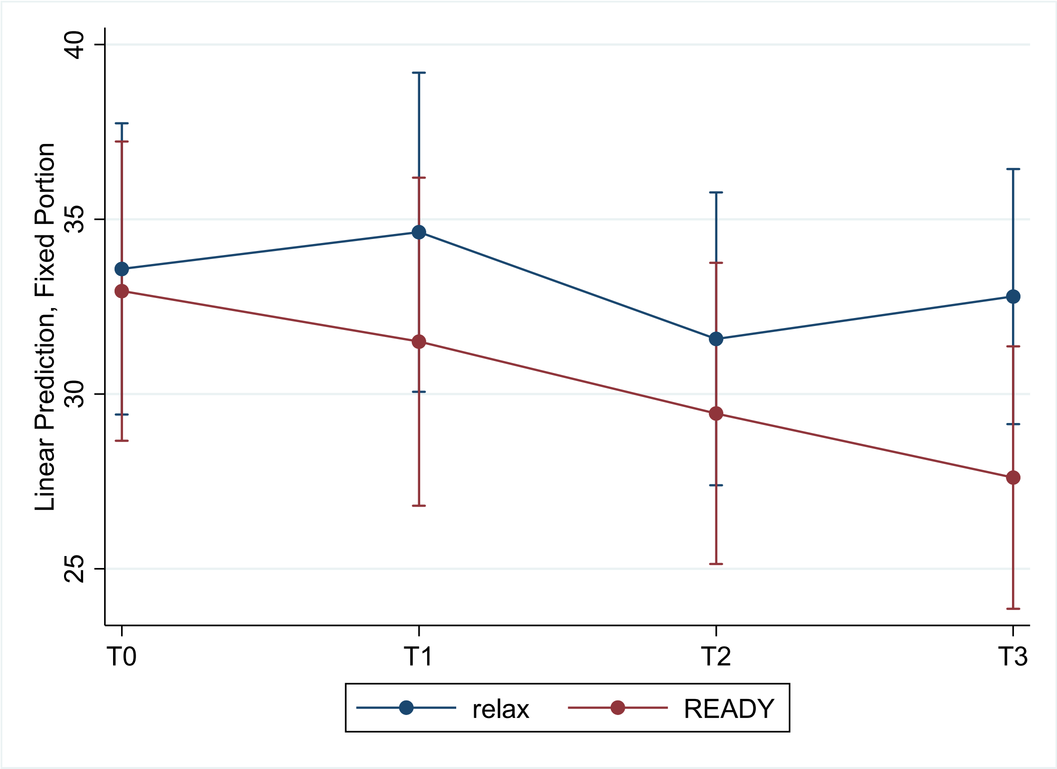

Linear Prediction, Fixed Portion
40
35
30
25
T0
T1
T2
T3
relax
READY

VLQ Total Score

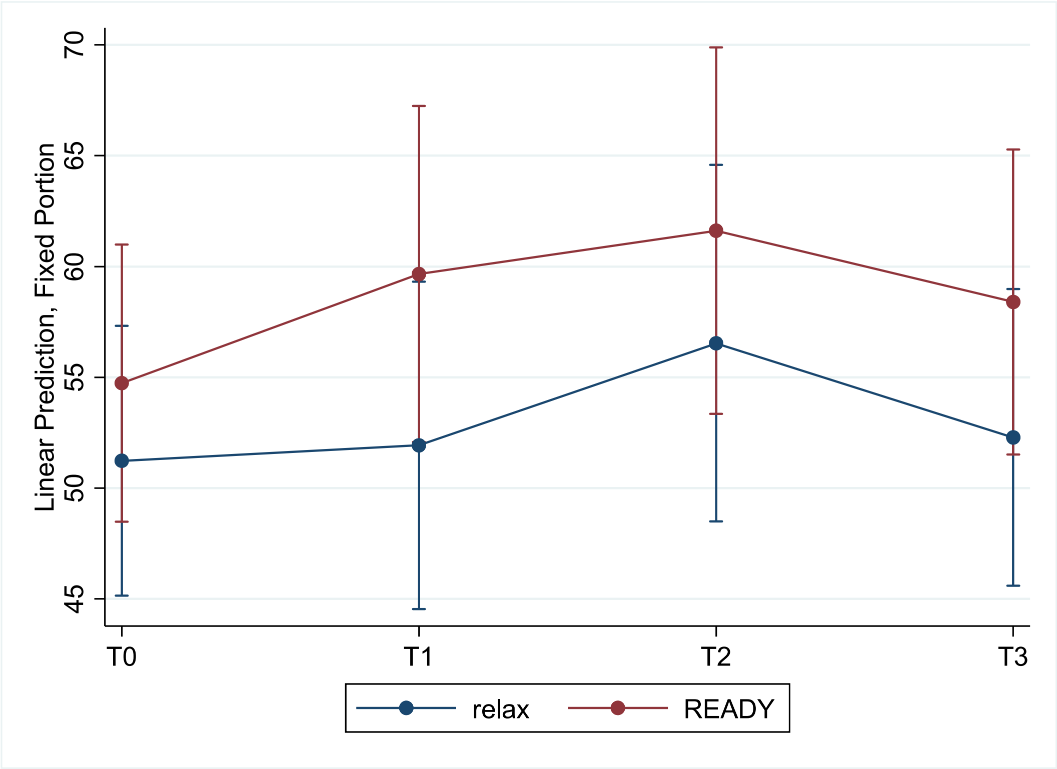

VLQ Importance

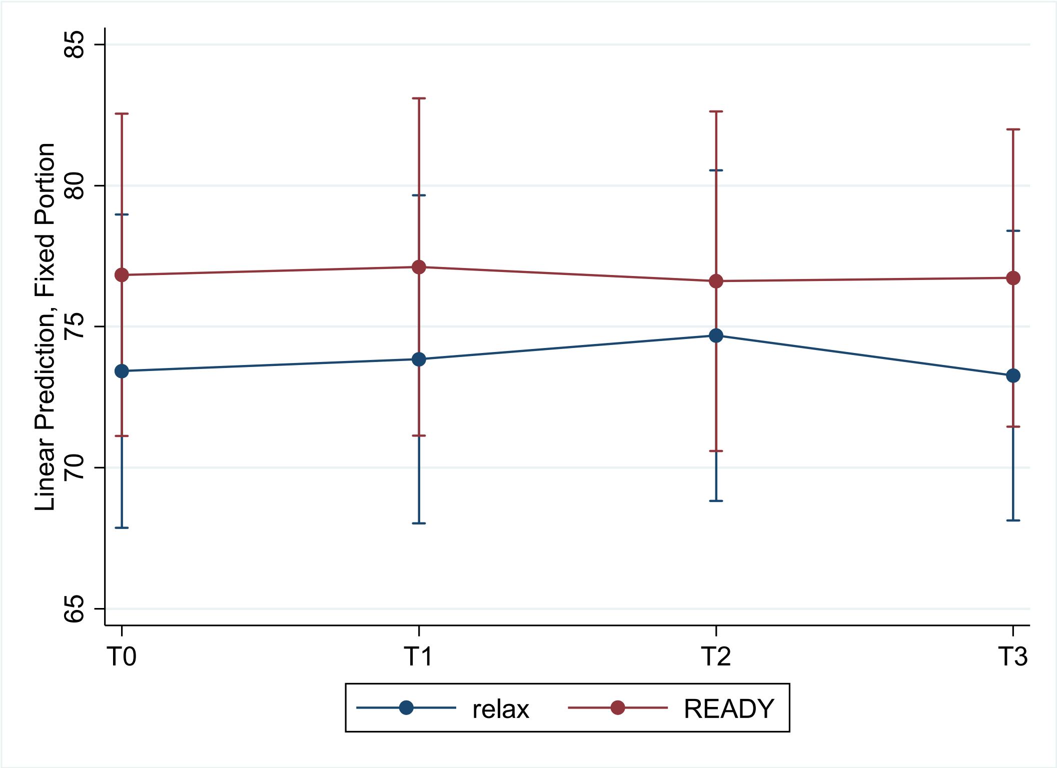

VLQ Consistency

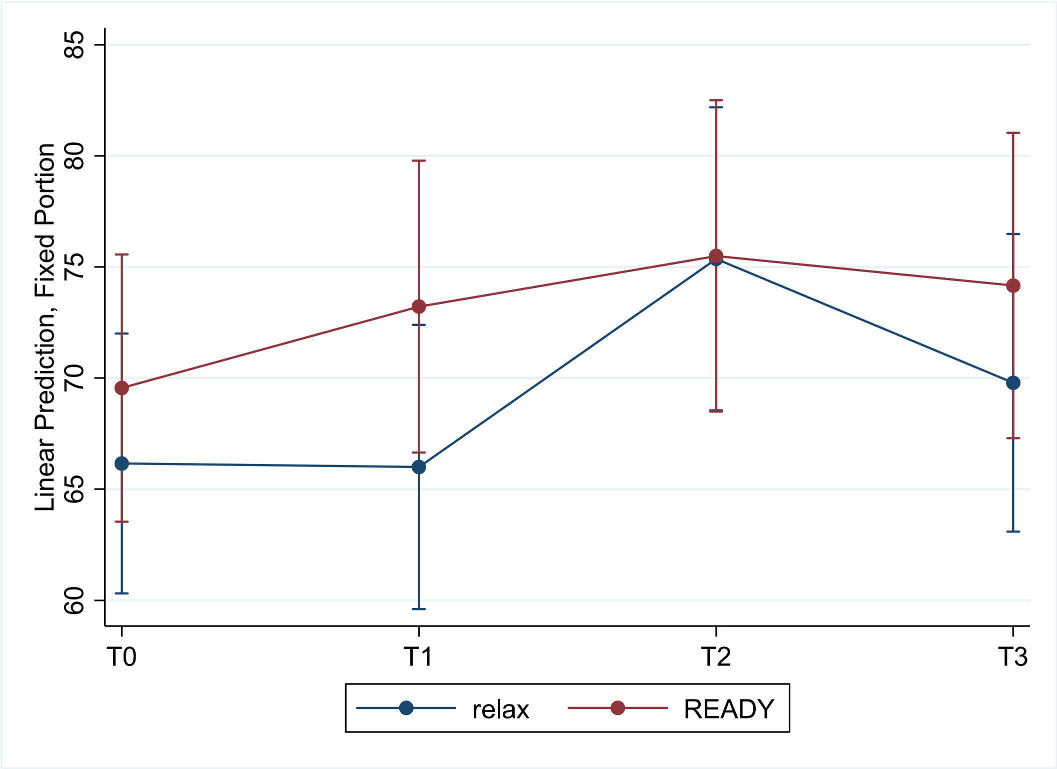

DDS

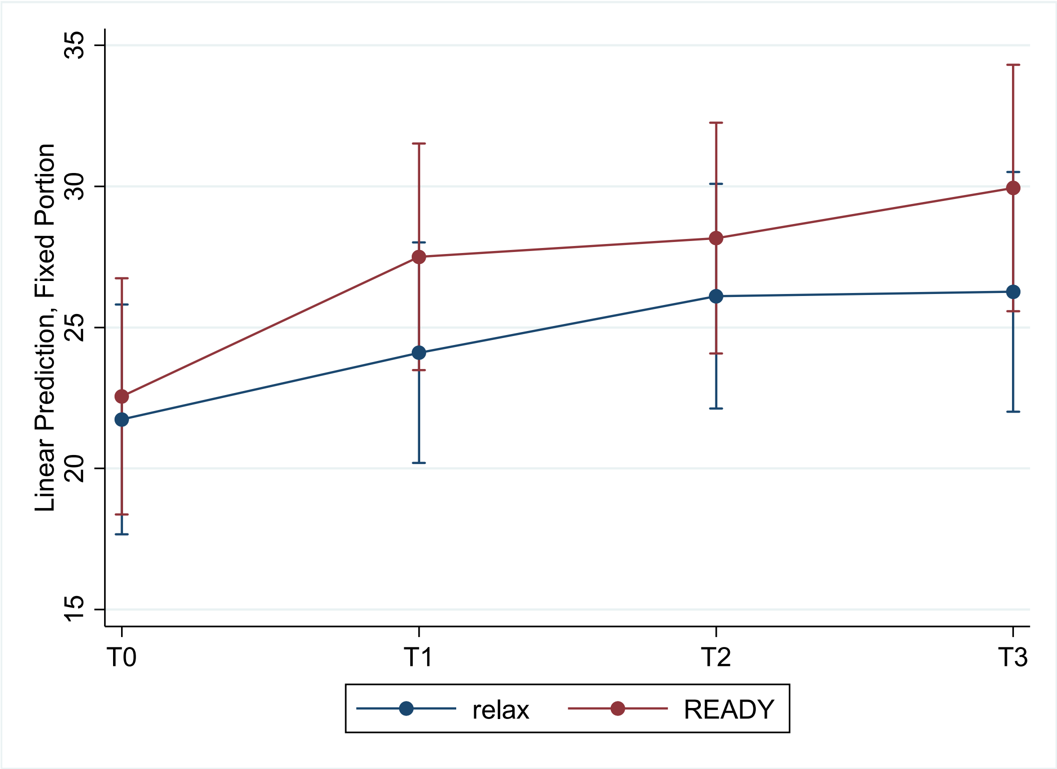

SEIQOL-DW

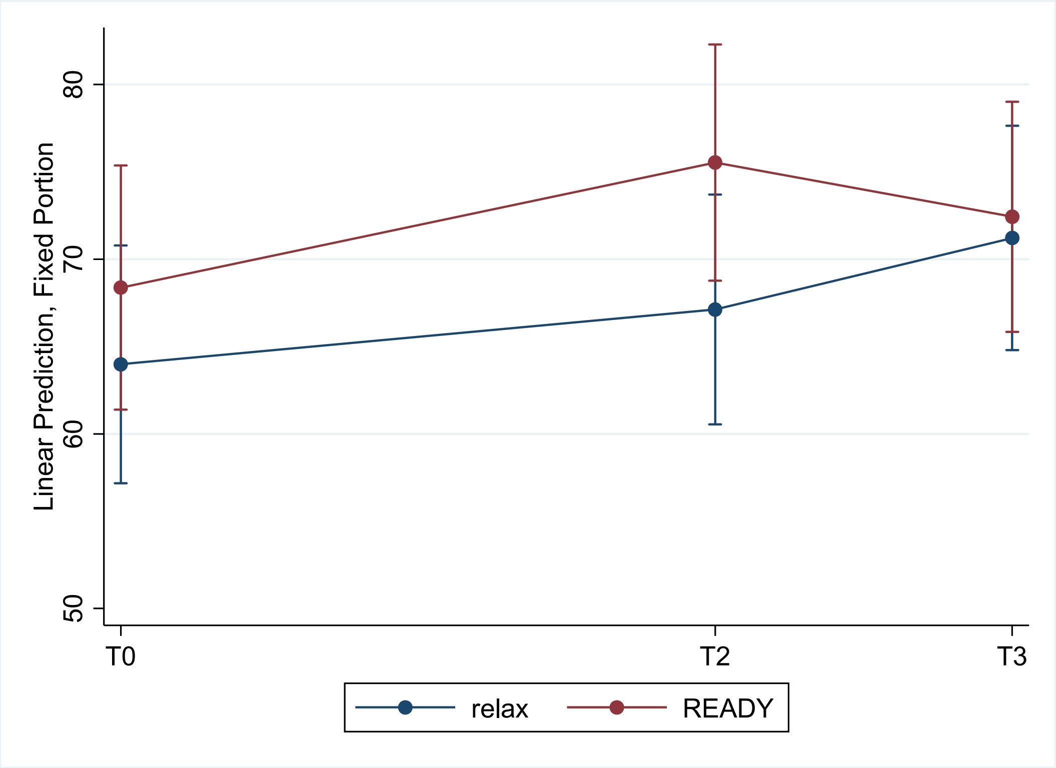
Linear Prediction, Fixed Portion
50
60
70
80
T0
T2
T3
relax
READY
