# Supplementary material for: A resilience group training program for people with multiple sclerosis: Results of a pilot single-blind randomized controlled trial and nested qualitative study
Source: PLoS One. 2020 Apr 9;15(4):e0231380. doi: 10.1371/journal.pone.0231380 (PMC7145197; doi:10.1371/journal.pone.0231380)
Supplement: S7 Appendix — (PDF) [file pone.0231380.s007.pdf]

S7 Appendix – Qualitative study quotes

Sociodemographic and clinical characteristics of the qualitative study sample, individually presented for each participant.

	MCC MSQOL-54 MHC	Age	Sex	Randomization	EDSS	Disease Duration	MS type	Education	Marital status	Employment status	Autonomy
P01	No	53	F	READY	1.5	3	RR	Degree	Single	Full time employed	Independent
P02	Yes	45	M	READY	6.5	32	SP	Degree	Married	Full time employed	Partial assistance
P03	Yes	39	F	READY	1.5	4	RR	Degree	Single	Full time employed	Independent
P04	Yes	59	F	READY	3.0	36	SP	Middle school diploma	Married	Retired	Partial assistance
P05	No	54	F	READY	2.0	28	RR	Degree	Married	Full time employed	Independent
P06	Yes	49	F	READY	2.0	25	RR	Degree	Married	Full time employed	Independent
P07	Yes	20	F	READY	0.0	5	RR	High school diploma	Single	Student	Independent
P08	Yes	33	F	READY	1.5	1	RR	Degree	Single	Unemployed	Independent
P09	Yes	52	M	READY	2.5	7	RR	PhD	Single	Freelance	Independent
P10	Yes	46	F	READY	1.0	14	RR	PhD	Married	Full time employed	Independent
P11	No	31	F	READY	2.0	5	RR	Degree	Single	Student	Independent
P12	Yes	53	F	READY	2.5	24	RR	Degree	Divorced	Full time employed	Independent
P13	No	53	M	READY	6.5	6	PP	Degree	Married	Full time employed	Partial assistance
P14	Yes	34	M	READY	1.0	5	RR	Degree	Single	Unemployed	Independent
P15	Yes	48	M	READY	6.0	11	SP	High school diploma	Married	Full time employed	Independent
P16	No	45	F	READY	2.0	5	RR	High school diploma	Married	Full time employed	Independent
P17	Yes	53	F	READY	2.0	34	RR	Middle school diploma	Single	Full time employed	Independent
P18	Yes	41	F	READY	1.5	2	RR	Degree	Married	Full time employed	Independent
P19	Yes	57	M	Relax + READY	1.5	3	RR	Degree	Married	Unemployed	Independent
P20	No	55	M	Relax + READY	1.5	3	RR	Middle school diploma	Married	Full time employed	Independent
P21	Yes	49	M	Relax + READY	4.0	9	RR	Middle school diploma	Married	Full time employed	Independent
P22	Yes	49	M	Relax + READY	4.0	17	RR	Degree	Married	Full time employed	Independent
P23	Yes	40	F	Relax + READY	1.5	10	RR	High school diploma	Married	Full time employed	Independent
P24	Yes	35	F	Relax + READY	0.0	2	RR	Degree	Married	Freelance	Independent
P25	Yes	57	M	Relax + READY	2.0	3	RR	Degree	Single	Unemployed	Independent
P26	No	51	M	Relax + READY	6.5	30	SP	Middle school diploma	Married	Retired	Partial assistance
P27	Yes	57	F	Relax + READY	3.0	30	RR	Middle school diploma	Married	Part-time employed	Independent
P28	No	28	F	Relax + READY	3.0	2	RR	Middle school diploma	Single	Unemployed	Independent
P29	Yes	51	M	Relax + READY	6.5	20	SP	High school diploma	Married	Unemployed	Partial assistance
P30	No	49	F	Relax + READY	1.5	1	RR	Degree	Single	Full time employed	Independent

Note. Age and disease duration are reported in years. MCC= Meaningful Clinical Change; RR= relapsing remitting; SP= secondary progressive; PP= primary progressive.

Theme 1 - Attitudes towards participation

Table a. Attitudes towards participation

**Curiosity**	*"I am very curious, particularly towards these techniques and new experiences. That's why I said: "why not!" when they offered me to participate. [P12: woman, 53 y, RR, EDSS 3.0, R]*
**Scepticism**	*"At the beginning I was sceptical about it" [P08: woman, 33 y, RR, EDSS 2.0, R]*
**Suspiciousness**	*"My first reaction was being suspicious!" [P12: woman, 53 y, RR, EDSS 3.0, R]*
**Looking for a group setting experience**	*"It was a while since I was looking for a group setting experience!" [P12: woman, 53 y, RR, EDSS 3.0, R]*
**Meeting other people with MS**	*"I was worried about meeting other people with MS" [P03: woman, 39 y, RR, EDSS 2.0, R]*
Desire	*"I was diagnosed when I was 15, but I had never had any contacts with the MS world before this project. Of course, I periodically met the neurologist and the psychologist, but I never met other people with MS. This time I felt the desire to meet other people with my disease." [P07: woman, 20 y, RR, EDSS 0.0, R]*
**Put yourself out there (openness to experience)**	*"I put myself out there" [P18: woman, 41 y, RR, EDSS 2.0, R]*
**It was a bet with myself**	*"It was a bet with myself; I've never participated in a group therapy before!" [P14: man, 34 y, RR, EDSS 1.0, R]*
**Dedicating time to yourself**	*"I chose this project with the purpose of dedicating time to myself!" [P16: woman, 45 y, RR, EDSS 2.0, R]*
**Dealing better with MS**	*"When I first thought about accepting this invitation my hope was to learn how to deal better with the disease" [P13: man, 53 y, PP, EDSS 7.0, R]*

Theme 2 - Perceptions of program composition

Table b. The program

READY		Relaxation	
Positive		*Positive*	
**Really helpful**	*"This experience has been enlightening! I was totally stuck I got totally beached on my past, thoughts and worries. I had no idea how to deal with my life. The READY program helped me in starting my life again!" [P18: woman, 41 y, RR, EDSS 2.0, R]*	**Really helpful**	*"I use relaxation techniques especially in my working activities. I am obtaining great results" [P27: woman, 57 y, RR, EDSS 3.0, r+R]*
**Different from other therapies**	*"I have already done psychotherapy, but this approach is really different, so unique! So much more practical! It really provides you with new skills." [P12: woman, 53 y, RR, EDSS 3.0, R]*	**Pleasant**	*"I really like the first program, I was happy to participate". [P26: man, 51 y, SP, EDSS 7.0, r+R]*
**Cutting-edge**	*"I would define READY a cutting-edge program! Particularly here in Italy!" [P09: man, 52 y, RR, EDSS 3.0, R]*		
**Time to dedicate to myself**	*"I dedicated time to myself; I wouldn't never done without this excuse!" [P03: woman, 39 y, RR, EDSS 2.0, R]*		
**Helps me to be open**	*"Now I'm really open to other initiatives! Please, don't exitate to contact me if you organize other similar initiatives! Call me!" [P23: woman, 40 y, RR, EDSS 2.0, r+R]*		
**Good example of integrating medical and psychological care**	*"When you have a disease, people think the most important thing is taking care about it under a physical point of you, with the consequence that you are alone in dealing with it. There is no attention to the psychological part. This is not only the case of MS, it is true for all the diseases. The medical intervention should always be integrating with a psychological one! You*		

	really need that and this is what I have found thanks to this experience!" [P16: woman, 45 y, RR, EDSS 2.0, R]		
**Should be integrated into hospital services**	*"I do believe the READY program should be included in the hospital service chart!" [P19: man, 57 y, RR, EDSS 2.0, r+R]*		
**Recommended for ill and non-ill persons**	*"Everybody would benefit from this program! I highly recommend it to everyone!" [P29: man, 51 y, SP, EDSS 7.0, r+R]*		
Negative		*Negative*	
NONE		**Not relaxing**	*"I didn't feel relaxed during the session… maybe because I am really rational... but that's it!" [P19: man, 57 y, RR, EDSS 2.0, r+R]*
		**A hard experience**	*"I am sorry to say that but it has been a hard experience! Time was running slow!" [P19: man, 57 y, RR, EDSS 2.0, r+R]*
		**Passive**	*"Compared to READY, relaxation was so passive!" [P24: woman, 35 y, RR, EDSS 0.0, r+R]*
		**Only an ice breaking activity**	*"In my opinion, relaxation has been an ice breaking activity, a way to create new connection" [P30: woman, 49 y, RR, EDSS 2.0, r+R]*

Table c. The format

READY		Relaxation	
**Ok as is**	*"I found the duration of the program and each session well-balanced" [P26: man, 51 y, SP, EDSS 7.0, r+R]*	**Ok as is**	*"Duration and frequency of the session were ok!" [P23: woman, 40 y, RR, EDSS 2.0, r+R]*
**Prefer longer program**	*"I wish it had lasted longer, we [group members] discussed about that and established that it would be great to increase the number of session from 7 to 10. You need some more sessions for ice braking." [P07: woman, 20 y, RR, EDSS 0.0, R]*<br><br>*"I wish each session could last longer to have more time for practicing and group work. Sometimes I wanted to repeat the same exercise with more than one person, but time was running out!" [P01: woman, 53 y, RR, EDSS 2.0, R]*	**Prefer shorter program**	*"I wished it [relaxation session] could finish fast" [P19: man, 57 y, RR, EDSS 2.0, r+R]*

Table d. The materials

READY		Relaxation	
**Useful**	*"Both the audio and the booklets were really useful! You may find everything we discussed during the session in the booklet! Really helpful!" [P07: woman, 20 y, RR, EDSS 0.0, R]*<br>*"Absolutely precious! I keep them* [ession booklets] *on my night stand! I consult them whenever I feel the need to." [P18: woman, 41 y, RR, EDSS 2.0, R]*	**Useful**	*"The audio file [name of the facilitator] gave to us were really useful!" [P24: woman, 35 y, RR, EDSS 0.0, r+R]*
**Commitment style**		-	-
High commitment during the program	*"I didn't practice hard between the sessions, but whenever I feel the need, I re-read the booklets or my notes looking for some suggestions!" [P26: man, 51 y, SP, EDSS 7.0, r+R]*		
High commitment during and after the program	*"I practiced the new strategies after each session, I was really dedicated!" [P03: woman, 39 y, RR, EDSS 2.0, R]*		
High commitment during the program and then decrease	*"I always did the homework and even now I consult the booklet again and practice! In this way, I can also be aware of my progress!" [P01: woman, 53 y, RR, EDSS 2.0, R]*		
Low commitment during the program but use materials after its conclusion	*"I found it helpful and I used them during the training, but now I'm not practicing very often" [P26: man, 51 y, SP, EDSS 7.0, r+R]*		
No commitment	*"I never did the homework! I've never been a good student, but I was really engaged during the session. Maybe I will re-read the booklet one day!" [P17: woman, 53 y, RR, EDSS 2.0, R]*		
**Importance of commitment style**		-	-
During the program	*"Good will is what counts! It is really important to dedicate space to practice what you learn during the sessions. Life can be frenetic, but sometimes we use that as an excuse!" [P03: woman, 39 y, RR, EDSS 2.0, R]*		
After the program	*It would be important to brush up on it! Practice from time to time is important, especially now that the READY is ended." [P03: woman, 39 y, RR, EDSS 2.0, R]*		
During and after the program	*The READY program is really intense, a full immersion! You really need time to elaborate all the stimuli received during and after the training. I also bought an ACT book!" [P07: woman, 20 y, RR, EDSS 0.0, R]*		

Theme 3 - Program impacts on life domains

Table e. Program impacts on life domains

READY		Relaxation	
**Daily living**	*"It [READY] really improved my quality of life, I mean… I learnt a different approach to my life and this is hugely affecting my daily living. [P15: man, 48 y, SP, EDSS 6.0, R]*	**-**	-
**MS**	*"Before this experience I couldn't even pronounce the words "multiple sclerosis", I didn't speak with anyone about it, I have never met anyone with MS before… but after the READY I went to the "Young people with MS congress" in Rome! Can you believe that!? The program has changed the way I think about and deal with MS. Previously, I avoided it as much as I could. Avoidance was exactly one of the topic of the program and I was so touched by that session. I was studying law while I was attending the program but my dream was becoming a medical doctor! Now I decided to try the admission test of medicine, even if I am still scared about studying my disease. Accepting fear let me to live my life fully!" [P07: woman, 20 y, RR, EDSS 0.0, R]*	**MS (only MRI management)**	*"I used relaxation techiniques during my last MRI, it helped me!" [P24: woman, 35 y, RR, EDSS 0.0, r+R]*
**Working/academic activities**	*"The impact was strong! I took the decision to significantly reduce the working hours in order to dedicate more energy to other aspects of my life! I am so happy about this decision." [P13: man, 53 y, PP, EDSS 7.0, R]*	**Working activities**	*"Relaxation help me in dealing better at work! I found my work really stressful and these techniques are useful for managing it"" [P27: woman, 57 y, RR, EDSS 3.0, r+R]*
**Relationships**	*"This experience changed my relationship with others, particularly with my relatives. Relationships are now more peaceful and less conflictual!" [P06: woman, 49 y, RR, EDSS 2.0, R]*	**Family**	*"Relaxation has a beneficial effect on my family relationship: I would define it a virtuous cycle!" [P23: woman, 40 y, RR, EDSS 2.0, r+R]*
**Self-care**	*"I learnt to value things that were important to me and work hard to pursue them! I learnt to dedicate time to myself, without feeling guilty. I found my good mood and my smile again. I learnt there are strategies I can use to recognize thoughts and worries, make a room for them and accept them before they drag me in the abyss! I feel I am more brave and flexible. Even at work,*	**Self-care**	*"I practice relaxation when I want to stay alone and take care of myself. I need and enjoy there moments so much!" [P27: woman, 57 y, RR, EDSS 3.0, r+R]*

	I am daring more!" [P18: woman, 41 y, RR, EDSS 2.0, R]		
**Self esteem**	*"This program increased my self-esteem." [P23: woman, 40 y, RR, EDSS 2.0, r+R]*	**-**	-
**Personal growth**	*"I found it difficult at the beginning, but then, it has been crucial for my personal growth." [P07: woman, 20 y, RR, EDSS 0.0, R]*	**-**	-
**-**	-	**Sleeping**	*"I use relaxation when I go to sleep" [P27: woman, 57 y, RR, EDSS 3.0, r+R]*
**-**	-	**Level of tension**	*"I went home deeply relaxed. Tension faded away after each session." [P30: woman, 49 y, RR, EDSS 2.0, r+R]*
**-**	-	**Anxiety**	*"I found relaxation really useful for my anxiety!" [P24: woman, 35 y, RR, EDSS 0.0, r+R]*

Theme 4 – Program active elements

Table f. Specific factors

READY		Relaxation	
-	-	**Autogenic training**	*"I put in place relaxation techniques at work. I found my work environment really hard, I use autogenic training daily and I reached good results!" [P27: woman, 57 y, RR, EDSS 3.0, r+R]*
**Psychological flexibility**		-	-
Present moment awareness (mindfulness)			
Formal practice	*"Practicing mindfulness is really helpful. It is not about relaxation, I can listen to the music and relax my body. When I practice mindfulness I am in contact with myself and the environment around me, it reminds me what it is important to me!" [P01: woman, 53 y, RR, EDSS 2.0, R]*		
Informal practice	*"Sometimes I wash the dishes listening to the sound of the water or I have a shower and I dedicate time to notice the different fragrances I can smell, the sensation of the water or the soap on my body, the foam, and the sounds when the water falls against the shower box or on my body. It may seem a stupid thing, but I really love doing it." [P04: woman, 59 y, SP, EDSS 3.0, R]*		
Openness to experience (Acceptance)	*"You need to let yourself feel all the shadows, whatever they are: pleasant or no! This is what I have learned and what I have done! Before READY I really had control on myself, but during a session I cried... It was unbelievable, I have given everything! When I let other people see my inner self, their reaction was warm and compassionate, I really felt emptiness flying away!" [P03: woman, 39 y, RR, EDSS 2.0, R]*		
Defusion	*"I am so much more effective now, because I am now able to get unstuck from my thoughts and stop telling me stories. I can observe them as if they were clouds in the sky." [P01: woman, 53 y, RR, EDSS 2.0, R]*		
Observing self	*"I found extremely useful learning how to observe myself, defusion and observing self techniques really have helped me!" [P25: man, 57 y, RR, EDSS 2.0, r+R]*		

Connection with personal values	*"During a mindfulness practice, I really had an intuition: "I have lost my time! I need to dedicate my energy to other things, I want to dedicate my life to what really mean to me! It was the session about values. It has really changed my life!" [P18: woman, 41 y, RR, EDSS 2.0, R]*		
Values driven committed actions	*"No matter how pervasive will be the disease, how hardly it will affect my body in the future; I learnt there is always the opportunity to express what it is important to me! Even though, I won't be able to move anything in the future I could always find a way to express myself!" [P09: man, 52 y, RR, EDSS 3.0, R]*		
**Social connectedness**	*"I opened up again to others; I am now willing to welcome different points of view. This new attitude helps me a lot! Relationships with other are now really important. It is not only about receiving or giving, is more about sharing my true self and being authentic! This totally change the quality of my connections." [P18: woman, 41 y, RR, EDSS 2.0, R]*	-	-
**Self-care**	*"You know what?! Participating in the READY program let me understand that I am also important, not only the other people! I learnt focusing on myself, my needs and taking care of myself. Sometimes I Have the thought "this is bad, you are selfish", but then I feel the importance of taking care of myself... and I can let this thought come and go." [P18: woman, 41 y, RR, EDSS 2.0, R]*	-	-
**Leisure time activities**	*"I learnt the importance of each single playful moment! There is always time for doing the laundry, cleaning up the house, or cooking. I learnt you can postpone the laundry, and having a pizza with your son instead, or having a coffee with a friend of yours, enjoying the moment together! This is totally new to me!" [P05: woman, 54 y, RR, EDSS 2.0, R]*	-	-

Table g. Nonspecific factors

READY		Relaxation	
**Group effect**		**Group effect**	
To see eye to eye	*"It was the first time that I had the courage to tell that going to the park, being able to run, is really important to me. I remember that day, I was looking down on the ground, and thinking about what I had just said was silly and inappropriate. But when I looked up again I saw all these people that were really supporting and understanding me! I had a warm feeling spreading through my heart and I felt really ok! In that very moment I thought: yes, I would do this experience again and again! It was worth it!" [P03: woman, 39 y, RR, EDSS 2.0, R]*	To see eye to eye	*"I think the effect is due to the group. Being part of a group in which people have my same feelings was really special. They can really understand me. It has been the first time I had the opportunity to have this kind of confrontation!" [P23: woman, 40 y, RR, EDSS 2.0, r+R]*
Sharing opinions and points of view	*"Since the day of the diagnosis I have had a lot of doubts and unanswered questions! Participating in this group, meeting several people that have the same problems and difficulties as me, opening up to different perspectives and points of view helped me a lot! [P18: woman, 41 y, RR, EDSS 2.0, R]*	Sharing opinions	*"It was hard to meet people with a more severe MS at the beginning. On one side, to recognize my MS was less sever comforted me, on the other side it makes me wonder about my condition in ten years! Dealing with this fear was important. I found the positive side of this experience." [P23: woman, 40 y, RR, EDSS 2.0, r+R]*
Feeling of "we-ness"	*"We are still in touch! We call and write each other, no matter if the program finished, there is still this great connections between us. We didn't loose it! If someone is dealing with something difficult we are all there, ready to write something and show our support! I keep feeling part of something special that goes beyond the program" [P04: woman, 59 y, SP, EDSS 3.0, R]*	-	-
Social support	*"Being part of a group called READY for SM helps a lot! If someone said "I'm at my worst" we all keep texting each other, maybe funny text. We are there for each other! It is like a strength generated from this experience that we cultivate daily. We are the READY group!" [P01: woman, 53 y, RR, EDSS 2.0, R]*	-	-
Group as reference point	*"Now I feel I have a reference point that I didn't have before! I felt so lonely before this experience, now I don't" [P05: woman, 54 y, RR, EDSS 2.0, R]*	-	-

Feeling of being cared for	*"Thanks to this kind of initiative you [the patient] have no more the feeling of being only a number in the doctor list!" [P09: man, 52 y, RR, EDSS 3.0, R]*	-	-
Meeting other people with MS	*"One of the most important thing of this experience was meeting other people with MS. We shared our experience and discovered we have to deal with the same difficulties, fears and the desire to find a way to live better and deal with the disease without ruining our life more than the MS is already doing." [P06: woman, 49 y, RR, EDSS 2.0, R]*	-	-
Meeting people with a more severe MS	*"It was a big surprise to meet people with a more severe MS and see how they deal with it. They really have the strength and capacity to overcome difficulties by accepting what they can't change and valuing what they can still do! This was the most important lesson I learned, one of my most valuable memory!" [P14: man, 34 y, RR, EDSS 1.0, R]*	Meeting people with a more severe MS	*"I am lonely. With the only exception of my parents I am always alone. Participating in this group, meeting other people with my diease sharing different opinion and points of view, this is the key! This is what really affected me!" [P26: man, 51 y, SP, EDSS 7.0, r+R]*
**Facilitator**			
Connection with the facilitators	*"We walked this path together, the facilitator was spectacular in promoting this sense of connection." [P01: woman, 53 y, RR, EDSS 2.0, R]*	-	-
Facilitation style	*"It was a great experience! The facilitator was one of us. I never felt her detached! We really enjoyed her professionalism mixed with friendship and affection. I think this is way the group and the experience went so well!" [P03: woman, 39 y, RR, EDSS 2.0, R]*	Faciliation style	*"I think she [the facilitator] created the perfect atmosphere. This really makes the difference!" [P20: man, 55 y, RR, EDSS 2.0, r+R]*
"Practice what you preach"	*"Her [the facilitator] tremendous enthusiasm was contagious. She is the living example of what she teaches during the program!" [P15: man, 48 y, SP, EDSS 6.0, R]*	-	-
**Personal factors**		**-**	**-**
Willingness	*"I think you need to be open to this experience. You can't obtain the desired outcome, if you are not willing to be open to what will happen" [P07: woman, 20 y, RR, EDSS 0.0, R]*		
Commitment	*"You must tell people that this program needs a personal commitment, before asking for a brush-up meeting, they need to practice what they learn during READY!" [P25: man, 57 y, RR, EDSS 2.0, r+R]*		

**-**	-	**Self-care**	*"Relaxation is not my thing! During the relaxation training I understood I would have never practiced at home. Hence, I decided to dedicate time to myself. Instead of practicing relaxation, I focused on self-care! I started doing sport, going out with friends, etc.. These activities were so much more satisfing than relaxation could be! Taking care of myself positively affected me." [P23: woman, 40 y, RR, EDSS 2.0, r+R]*

Theme 5 – Program improvements trajectories

Table h. Program improvements trajectories

READY		Relaxation + READY	
**Illumination and further strengthening**	*"I started perceiving some changes during the program. The light bulb came on during a session and then I started consolidating it day by day. It's like a deep personal change; nobody can steal it from you once you have it." [P06: woman, 49 y, RR, EDSS 2.0, R]*	**-**	-
**Improvement and further decrease**	*"The effect was stronger at the beginning, then it begun to slip as I went back to my life. I recognize I have more tools now, a "positive toolbox" for my future." [P03: woman, 39 y, RR, EDSS 2.0, R]*	**Improvement and further decrease**	*"I was really involved and activated by the program during the sessions. I practiced and use all the techniques more. Now that it is finished is fading away a little bit." [P24: woman, 35 y, RR, EDSS 0.0, r+R]*
**Progressive improvement during and after the training**	*"It is like planting a seed that grows up day by day." [P10: woman, 46 y, RR, EDSS 1.0, R]*	**Progressive improvement since deciding to participate in the project**	*"It started with the decision to take part to the project. It increases with relaxation mainly for the group effect, not the technique and it increases hugely with READY thanks to both techniques and a more active interaction with the group." [P23: woman, 40 y, RR, EDSS 2.0, r+R]*
**The improvement can be recognized after the training**	*"They [improvements] happen during the training, but to be honest I became aware of them when the program was completed. I start noticing them this summer. Things in my life have started taking new directions, but it is only now that I can say I am sure it is thanks to my participation to READY." [P01: woman, 53 y, RR, EDSS 2.0, R]*	**-**	-

**N/A**	N/A	**Progressive improvement during and after the programs with a particular insights during READY**	*"A lot of changes happened while I was doing this experience, particularly during the READY program. Other aspects appeared or were consolidated after it was concluded. It's a work in progress, I really want to cultivating this richness more and more." [P27: woman, 57 y, RR, EDSS 3.0, r+R]*
		Relaxation facilitated the creation of the group, which READY capitalized on with skill development	*"The first program [relaxation] let us to create a connection between and the feeling of being part of a group. It really facilitate the READY. Relaxation allow you to let your tension go, the READY program instead, it is more about the techniques. The first part is about building up the group, the second one really teach you new concepts and new skills." [P29: man, 51 y, SP, EDSS 7.0, r+R]*
		The READY is more effective if you do the relaxation before it	*"You work better during READY if you have already done relaxation. Definitely!" [P29: man, 51 y, SP, EDSS 7.0, r+R*

Theme 6 - Program differences and similarities

Table i. Superiority of one program over the other

READY superior to relaxation		Relaxation superior to READY	
**Higher satisfaction**		**Skills acquisition is easier**	*"Learning READY skills is harder than relaxation. It was easier with relaxation technique." [P24: woman, 35 y, RR, EDSS 0.0, r+R]*
More pleasant	*"I enjoyed READY more than relaxation because it has lots of variety, you learn so much more things, you can share your experience with other participants, even really intimate ones!" [P19: man, 57 y, RR, EDSS 2.0, r+R]*		
More engaging	*"The READY program was more engaging than relaxation. The first program [relaxation] was really passive" [P24: woman, 35 y, RR, EDSS 0.0, r+R]*		
More interesting	*"The two experiences are totally different. I would define relaxation an ice breaking activity, a way to meet other people. It wasn't really innovative. The READY program is a totally different thing. We received what I call "the pills": new skills and techniques!"[P30: woman, 49 y, RR, EDSS 2.0, r+R]*		
More useful	*"I didn't benefit so much from relaxation. With the READY, it was different! I recognized the importance of mindfulness, of all the psychological flexibility five domains, and resilience! This concept is so much clearer now! Now I am able to understand each of the five domains, recognize my warning signs and strength my inner strength. This is why I found this program really useful!" [P19: man, 57 y, RR, EDSS 2.0, r+R]*		
More detailed and deeper	*"We went deeper and deeper during the READY program in to our inner self. This is what I needed! I really appreciate being more in contact with the present moment!" [P23: woman, 40 y, RR, EDSS 2.0, r+R]*		
More involving	*"I noticed we were more engaged and involved during READY. The connection beween us was stronger during READY than relaxation." [P28: woman, 28 y, RR, EDSS 3.0, r+R]*		
More self-disclosure	*"We opened up during READY, really more than during relaxation. We put our feelings down and had great insights. We understood things we never did!" [P23: woman, 40 y, RR, EDSS 2.0, r+R]*		
Richer topics	*"The first program [relaxation] was a bit "empty". Something really filled up this experience when we started READY. We received a lot of information, practiced so many strategies, This experience was so heaped!" [P20: man, 55 y, RR, EDSS 2.0, r+R]*		
More active	*"READY is more interesting than relaxation*		

	because I feel more active and engaged than relaxation. Honestly, I love to be in action!" [P23: woman, 40 y, RR, EDSS 2.0, r+R]		
Better setting	*"We got in circle during READY, this aspect provide us with a great sense of closeness and warmness. It facilitates the connections, more openness and sharing." [P23: woman, 40 y, RR, EDSS 2.0, r+R]*		
I keep practicing only READY strategies	*"I must keep cultivating these new skills. Sometimes I dedicate time to review my session notes and read the booklet, or I practice mindfulness. I am not really constant, but I can recognize the validity and I want to be more committed." [P19: man, 57 y, RR, EDSS 2.0, r+R]*		
I would suggest only READY	*"I have already told you, you should activate a service to provide READY as a routine intervention in the hospital, available not only for people with MS, but also other conditions. Moreover, it would be great if it would also be offered to patients' relatives" [P19: man, 57 y, RR, EDSS 2.0, r+R]*		
**Impact on more life areas**			
Daily living	*"Thanks to the READY program you learn a different way to deal with your life! I can't say the same with relaxation" [P24: woman, 35 y, RR, EDSS 0.0, r+R]*		
MS	*"The READY program made me feel more confident on my inner resource. As a consequence I started thinking about MS in a different way!" [P21: man, 49 y, RR, EDSS 4.0, r+R]*		
Working activities	*"I have used all the things I learnt during READY quite a lot, particularly at work!" [P27: woman, 57 y, RR, EDSS 3.0, r+R]*		
Family	*"Thanks to READY I have paid more attention to my family and my kids." [P21: man, 49 y, RR, EDSS 4.0, r+R]*		
Self-efficacy	*"It [READY] also increased my self-efficacy!" [P21: man, 49 y, RR, EDSS 4.0, r+R]*		
Self esteem	*"It [READY] really had a huge impact on me! I can really appreciate its effect on my self-esteem" [P27: woman, 57 y, RR, EDSS 3.0, r+R]*		
Personal balance	*"I found my balance thanks to this program. Not 100% but 70-80%! It is a great result to me and it is due to READY. We worked on the resilience shield, on the daily issues, we focused on values, on self-care and flexible perspectives!" [P27: woman, 57 y, RR, EDSS 3.0, r+R]*		
**Acquisition of more skills**			

Present moment awareness	*"This morning I was walking here for the interview. I noticed some beautiful flowers, they were just bloomed. I stopped there, in front of the tree and I enjoyed this marvellous show! I think this is part of the endowment of knowledge of the READY program." [P25: man, 57 y, RR, EDSS 2.0, r+R]*		
Openness to experience	*"I have increased acceptance about the disease and my life in general" [P22: man, 49 y, RR, EDSS 4.0, r+R]*		
Defusion	*"When the READY began, I was living a stressful situation. It was really hard to deal with it. Problems don't disappear, but now I am brave enough to deal with it. I have the courage to say: "No, wait a moment" and looking at the thoughts that scared me I can treat them like a movie on the TV, I can squeeze it, change the size, turn it down. In the end, you can even laugh! You can't change the reality, but it really helps you in playing down things!" [P25: man, 57 y, RR, EDSS 2.0, r+R]*		
Connection with personal values	*"Thanks to the READY I've valued more my family, particularly my husband!" [P27: woman, 57 y, RR, EDSS 3.0, r+R]*		
Values driven committed actions	*"Thanks to this program [READY] I took a different perspective: Why shouldn't' do something that is important to me even alone?! Should I really need someone else to do what I like the most? No! I now chose to do the things I love even by myself. If someone want to join me it is ok, but I do that anyway! Before this experience, it was totally different!" [P27: woman, 57 y, RR, EDSS 3.0, r+R]*		

Table l. Programs similarities

**Shared strengths**	
Pleasant	*"It was a wonderful experience! I would do both READY and relaxation again." [P21: man, 49 y, RR, EDSS 4.0, r+R]*
Useful	*"I was so happy after this experience! It really helped me. It was a positive experience. It helped me in dealing different with my life." [P21: man, 49 y, RR, EDSS 4.0, r+R]*
Appropriate format	*"Programs duration was ideal! I can't see any differences between the two programs about this aspect." [P23: woman, 40 y, RR, EDSS 2.0, r+R]*
Appropriate materials	*"Booklets and audio were really useful. There was a correspondence between what we discussed during the session and the materials provided." [P24: woman, 35 y, RR, EDSS 0.0, r+R]*
Acquisition of new strategies	*"Both READY and relaxation helped me. They provided you with different methods and strategies to deal better with the disease." [P28: woman, 28 y, RR, EDSS 3.0, r+R]*
I would recommend them	*"I would suggest both the programs!" [P24: woman, 35 y, RR, EDSS 0.0, r+R]*
Group effect	*"In both the programs… the possibility to share our experience with other people and better understand your own point of view. If you are alone, your point of view is narrow, buti f you can speak with other people, if you listen to them, you can really change your perspective! There are always different sides!" [P29: man, 51 y, SP, EDSS 7.0, r+R]*
Meeting other PwMS	*"This experience gave me the opportunity to meet other people with MS, sometimes with different level of difficulties. This has been a crucial aspect of this experience!" [P20: man, 55 y, RR, EDSS 2.0, r+R]*
**Shared weaknesses**	
Programs format	
Sessions interval	*"Sometimes waiting one week for the next session was too much! Time running fast during the session… I couldn't wait for the next session to arrive!" [P20: man, 55 y, RR, EDSS 2.0, r+R]*
Prolong the programs by increasing session	
Interval	*"Maybe you could also think to increase the time between sessions: once every two weeks in order to make the program last longer. This connection with the group and the facilitator is really precious. I wish we could enjoy it for more time." [P23: woman, 40 y, RR, EDSS 2.0, r+R]*
Number	*"It was such a beautiful experience that we were so sad when it finished! For this reason I would suggest to extend the number of the session… maybe it is not really necessary, but we all would like the program could last longer." [P20: man, 55 y, RR, EDSS 2.0, r+R]*
Suboptimal session time	*"The time of the session wasn't perfect. You can deal with that, but if I can change something… this is the only thing I would modify. Maybe late in the afternoon." [P23: woman, 40 y, RR, EDSS 2.0, r+R]*

Table m. Lack of distinction between READY and relaxation

**Difficulties in distinguishing between the two programs**	
Programs on a continuum	*"I can't split up the two experiences. We passed from one experience to the other one. I embraced this experience all together, as a whole." [P27: woman, 57 y, RR, EDSS 3.0, r+R]*
Confusion between relaxation and mindfulness	*"The relaxation/mindfulness program… I don't divide them. I think mindfulness and relaxation are really close to each other. To me relaxation means practicing mindfulness." [P25: man, 57 y, RR, EDSS 2.0, r+R]*
**Impact described as general effect of participating in the project**	
Daily living	*"Before this experience I shut down completely. I was ashamed of my condition. I avoided their gaze, or their questions. Now I totally open up to the world. I am dealing differently with life every day". [P29: man, 51 y, SP, EDSS 7.0, r+R]*
MS	*"This group experience really facilitate speaking about the disease. Before them I have never talked anybody about it. Eventually, I subscribed some groups on facebook. Now I can easily open up to people! I think this is an effect of being in a group!" [P23: woman, 40 y, RR, EDSS 2.0, r+R]*
MRI	*"I have fear of the MRI, but now I use some technique to stay more calm and relaxed." [P24: woman, 35 y, RR, EDSS 0.0, r+R]*
Working activities	*"I am now dealing with a difficult situation at work. This project has helped me. II remember to myself that yes, job is an important area of my life, but it is not everything! Moreover, if you deal with the situation with calm, no panic, you can find the way". [P23: woman, 40 y, RR, EDSS 2.0, r+R]*
Relationship	*"Now I am more open to new experience of integrated care. I totally shut down because of the fear of dealing with everything... the neurologist, my fiends… Instead, thanks to this experience, I found myself more comfortable in every relationship." [P29: man, 51 y, SP, EDSS 7.0, r+R]*
Patient-neurologist relationship	*"Now I am more open to new experience of integrated care." [P29: man, 51 y, SP, EDSS 7.0, r+R]*
Personality	*"I have always been really cerebral and rational, really negative over everything! After this experience I changed a lot... a giant leap for me." [P23: woman, 40 y, RR, EDSS 2.0, r+R]*
Openness to the experience	*"Meeting other people, opening up to them, sharing your thoughts and feelings, being able to relax yourself change the way you relate towards the world in general, your friends and dayly life activities. Going to the market may become a different way to share an experience with someone" [P29: man, 51 y, SP, EDSS 7.0, r+R]*
Reducing panic attacks	*"Now I panic less, while before this experience I lost it down so often!" [P27: woman, 57 y, RR, EDSS 3.0, r+R]*

Theme 7 – Suggested READY improvements

Table n. Suggested READY improvements

**Program format and materials**	
Adding booster/Recall online	*"It would be really useful to add another booster session. I noticed not everybody has the perseverance to keep cultivating this personal journey" [P09: man, 52 y, RR, EDSS 3.0, R]*
Increase number of sessions	*"At the beginning, 7 sessions seem enough or even too much, but once you started you really want it to last longer. I would have added more sessions to consolidate what you learnt!" [P06: woman, 49 y, RR, EDSS 2.0, R]*
Increase duration of sessions	*"It's like going to primary school, once you learn reading and writing you can use the textbook, but I would have really appreciated to have more time for assimilating and practicing during each session!" [P01: woman, 53 y, RR, EDSS 2.0, R]*
Increase time between sessions	*"More time between one session and the other in order to have more time for practicing." [P01: woman, 53 y, RR, EDSS 2.0, R]*
Digitalize the materials	*"I would very much appreciate to have the same materials but in a digital format. Sometimes the booklet seems more like a textbook… as when you were at school and you had homework to do. If these materials were more coloured or cools, they would probably attracted more." [P08: woman, 33 y, RR, EDSS 2.0, R]*
A dedicated repository with real life models of READY skills application	*"I would like to access to a repository containing a series of positive application of the skills we learned during the READY program. A sort of database with good examples of READY skills application to daily life." [P13: man, 53 y, PP, EDSS 7.0, R]*
**Setting**	
Physical Setting	
Outside the MS Center	*"What about organize the session outside the hospital, maybe in some public space?!" [P13: man, 53 y, PP, EDSS 7.0, R]*
Quieter room	*My suggestion is to run the session in a quiter room and to use a microphone for the guided meditation. The facilitator voice was amazing, but low. Sometimes it was hard to properly hear." [P01: woman, 53 y, RR, EDSS 2.0, R]*
Group composition	
Age	*"I was the youngest, all the others participants were older… more than 17 years older. We were in different phases of our life. I would very much appreciated to have someone with my age. To be totally honest, sharing this experience with older people was also good and really useful"[P07: woman, 20 y, RR, EDSS 0.0, R]*
Disease severity	*"One aspect that I would improve is more homogeneity in terms of gender, disease severity. I am not asking for a complete homogeneity. I needed at least one person I could feel close to in terms of these characteristic. I was the only male and the one with the most severe condition." [P02: man, 45 y, SP, EDSS 7.0, R]*
Gender	*I would have balanced the number of male and female in the group [P02: man, 45 y, SP, EDSS 7.0, R]*
Bigger group	*"My suggestion is to organize bigger group, at least 12 members for each group" [P07: woman, 20 y, RR, EDSS 0.0, R]*
Individual session with the facilitator	*"The group setting was a strength, definitely! However, I would have very much appreciated an individual session with the psychologist" [P08: woman, 33 y, RR, EDSS 2.0, R]*

**Topics**	
Add new topics	*“I would like to add other sessions in which we can explore new topics and once in which freely share our experience” [P05: woman, 54 y, RR, EDSS 2.0, R]*
Expand on current topics	*“Considering all the work we have done, I feel the need to be looked into further.” [P06: woman, 49 y, RR, EDSS 2.0, R]*
**Procedure for enrolling patients**	
Pre-program interview with facilitator	*“I felt like I was plunged into this group of strangers! I haven’t even met the facilitator before starting the program. My suggestion is to Planning an individual meeting with the facilitator before the beginning of the intervention.” [P11: woman, 31 y, RR, EDSS 2.0, R]*
Offering READY at the beginning of the patients’ health care pathway	*“When you first arrive at the MS centre you feel disoriented, you should offer the READY immediately.” [P07: woman, 20 y, RR, EDSS 0.0, R]*
**Additional meetings**	
For members across groups	*“You know what?! I would be really curious to meet the patients that participated in the other READY groups, to stay together and sharing our experience about the project and about the disease” [P16: woman, 45 y, RR, EDSS 2.0, R]*
For presenting study results	*“Could you please organize a meeting to present the study results?” [P14: man, 34 y, RR, EDSS 1.0, R]*
For family of people with MS	*“After this experience I would like to share more with my family. It would be wonderful if you could organize one or more meetings dedicated to families in a very informal style.” [P23: woman, 40 y, RR, EDSS 2.0, r+R]*
Offer READY to patients’ significant others	*“You should offer a READY for MS group to our family members. After this course my awareness has increased and sometimes I have the feeling the distance between me and my husband too.” [P05: woman, 54 y, RR, EDSS 2.0, R]*
